# Feasibility and Potential of Transcriptomic Analysis Using the NanoString nCounter Technology to Aid the Classification of Rejection in Kidney Transplant Biopsies

**DOI:** 10.1097/TP.0000000000004372

**Published:** 2022-10-27

**Authors:** Hilal Varol, Angela Ernst, Iacopo Cristoferi, Wolfgang Arns, Carla C. Baan, Myrthe van Baardwijk, Thierry van den Bosch, Jennifer Eckhoff, Ana Harth, Dennis A. Hesselink, Folkert J. van Kemenade, Willem de Koning, Christine Kurschat, Robert C. Minnee, Dana A. Mustafa, Marlies E.J. Reinders, Shazia P. Shahzad-Arshad, Malou L.H. Snijders, Dirk Stippel, Andrew P. Stubbs, Jan von der Thüsen, Katharina Wirths, Jan U. Becker, Marian C. Clahsen-van Groningen

**Affiliations:** 1 Department of Pathology, University Medical Center Rotterdam, Rotterdam, The Netherlands.; 2 Erasmus MC Transplant Institute, University Medical Center Rotterdam, Rotterdam, The Netherlands.; 3 Institute of Medical Statistics and Computational Biology, University Hospital of Cologne, Cologne, Germany.; 4 Department of Pathology, Clinical Bioinformatics Unit, Erasmus MC, University Medical Center Rotterdam, Rotterdam, The Netherlands.; 5 Department of Surgery, Division of HPB & Transplant Surgery, University Medical Center Rotterdam, Rotterdam, The Netherlands.; 6 Cologne Merheim Medical Center, Cologne General Hospital, Cologne, Germany.; 7 Department of Internal Medicine, Division of Nephrology and Transplantation, University Medical Center Rotterdam, Rotterdam, The Netherlands.; 8 Department of General Visceral Cancer and Transplant Surgery Transplant Center Cologne, University of Cologne Faculty of Medicine and University Hospital of Cologne, Cologne, Germany.; 9 Department of Pathology, Tumor Immuno-Pathology Laboratory, Erasmus University Medical Center, Rotterdam, The Netherlands.; 10 Department II of Internal Medicine and Center for Molecular Medicine Cologne, University of Cologne, Faculty of Medicine and University Hospital Cologne, Cologne, Germany.; 11 Department of Pathology, Academic Medical Center, Amsterdam, The Netherlands.; 12 Department of Internal Medicine, Faculty of Medicine, University Bonn, Bonn, Germany.; 13 Institute of Pathology, University Hospital of Cologne, Cologne, Germany.; 14 Institute of Experimental Medicine and Systems Biology, RWTH Aachen University, Aachen, Germany.

## Abstract

Transcriptome analysis could be an additional diagnostic parameter in diagnosing kidney transplant (KTx) rejection. Here, we assessed feasibility and potential of NanoString nCounter analysis of KTx biopsies to aid the classification of rejection in clinical practice using both the Banff-Human Organ Transplant (B-HOT) panel and a customized antibody-mediated rejection (AMR)–specific NanoString nCounter Elements (Elements) panel. Additionally, we explored the potential for the classification of KTx rejection building and testing a classifier within our dataset.

**Methods.:**

Ninety-six formalin-fixed paraffin-embedded KTx biopsies were retrieved from the archives of the ErasmusMC Rotterdam and the University Hospital Cologne. Biopsies with AMR, borderline or T cell–mediated rejections (BLorTCMR), and no rejection were compared using the B-HOT and Elements panels.

**Results.:**

High correlation between gene expression levels was found when comparing the 2 chemistries pairwise (r = 0.76–0.88). Differential gene expression (false discovery rate; *P* < 0.05) was identified in biopsies diagnosed with AMR (B-HOT: 294; Elements: 76) and BLorTCMR (B-HOT: 353; Elements: 57) compared with no rejection. Using the most predictive genes from the B-HOT analysis and the Element analysis, 2 least absolute shrinkage and selection operators–based regression models to classify biopsies as AMR versus no AMR (BLorTCMR or no rejection) were developed achieving an receiver-operating–characteristic curve of 0.994 and 0.894, sensitivity of 0.821 and 0.480, and specificity of 1.00 and 0.979, respectively, during cross-validation.

**Conclusions.:**

Transcriptomic analysis is feasible on KTx biopsies previously used for diagnostic purposes. The B-HOT panel has the potential to differentiate AMR from BLorTCMR or no rejection and could prove valuable in aiding kidney transplant rejection classification.

## INTRODUCTION

Histologic classification of a for-cause kidney transplant biopsy can encounter several challenges such as limited amount of material, limited reproducibility, and differential diagnostic dilemmas.^1,2^ This is particularly true for antibody-mediated rejection (AMR).^3^ Transcriptome analysis may be a solution for these limitations. In 2013, molecular diagnostics were first introduced in the setting of AMR.^4^ Several smaller studies have identified transcripts particularly useful for the diagnosis of AMR and different groups developed AMR-specific molecular panels of various sizes.^5-8^

Probably the most advanced strategy for transcriptome analysis in kidney transplants is the Molecular Microscope Diagnostic System (MMDx). The diagnostic use of MMDx for both AMR and T cell–mediated rejection (TCMR) classification has been validated in multicentric prospective trials.^9,10^ Additionally, the MMDx transcriptome analysis identified different disease pathways and major cellular components contributing to rejection. Although the MMDx system provides a commercially available solution for transcriptome-based diagnostics, the transplant community has not wholeheartedly adopted this solution.

For centers that are not using MMDx, the translation of molecular diagnostics into clinical practice faces several obstacles, such as numerous and partly overlapping gene sets and multiple different molecular diagnostic panels on which these gene sets have been analyzed, for example, microarray expression analysis and quantitative real-time polymerase chain reaction (qRT-PCR). There is currently no consensus on which gene sets and panels are preferred for differentiating between AMR, TCMR, and no rejection.^11^

The NanoString nCounter platform is an alternative methodology to microarray gene expression analysis (eg, the MMDx) and qRT-PCR for investigating the mechanism/category of transplant rejection. The NanoString nCounter assay and analysis have several advantages, including a required small amount of mRNA from formalin-fixed paraffin-embedded (FFPE) biopsies (100 ng), which is complementary to standard histomorphologic work-up, thus eliminating the need for an additional biopsy core. Additionally, mRNA target molecules are detected without a reverse transcription step, omitting the introduction of variability and allowing analysis to be performed within just 2 d, parallel to the histologic evaluation. Moreover, NanoString nCounter gene expression analysis is more sensitive than microarray analysis and equally sensitive as qRT-PCR.^12^ This permits bulk transcriptome analysis on left-over tissue from the same FFPE block that was used for conventional histopathologic diagnosis. In 2015, the Banff Molecular Diagnostics Working Group recommended the formation of molecular consensus gene sets as classifiers that associate with the main clinical phenotypes of TCMR and AMR.^13^ As a result, in 2017, a gene set was proposed that could be analyzed on different molecular panels.^11^ The commercially available Banff-Human Organ Transplant (B-HOT) panel was developed from a collaboration between NanoString and members of the Banff Foundation for Allograft Pathology. This B-HOT panel was specifically developed to analyze solid organ transplantation tissue, including kidney, lung, heart, and liver, and omits the need for centralized molecular profiling.^14^ At the Banff meeting in 2019, the commercially available B-HOT panel was introduced for its use within the NanoString nCounter platform.^14^ However, the use of the NanoString technology and the B-HOT panel to aid kidney transplant diagnosis and classification has yet to be investigated to take a step toward the implementation of molecular pathology in everyday clinical practice. Technical aspects of this technology (eg, normalization) need to be investigated to fulfill its potential. Moreover, the potential for kidney transplant rejection diagnosis and classification of gene expression patterns obtained from NanoString B-HOT panel analysis of FFPE kidney transplant biopsies still needs to be explored in a real-life setting, beyond the theoretical framework that underlies its design.

In this study, we explored the feasibility and potential of NanoString nCounter gene expression analysis as a supporting tool for kidney transplant rejection diagnosis and classification. To achieve this, we (a) identified the most stable reference transcripts for NanoString gene expression analysis of FFPE kidney transplant tissue; (b) reassessed the reproducibility of the NanoString assay on kidney transplant biopsies in clinical practice; (c) investigated gene expression patterns of kidney transplant biopsies without a diagnosis of rejection and with a diagnosis of AMR or TCMR using the NanoString B-HOT panel and a custom NanoString nCounter Elements (Elements) AMR-specific panel; (d) explored the potential of NanoString gene expression analysis of kidney transplant biopsies for the diagnosis and classification of kidney transplant rejection building 2 classifiers that include the most predictive genes from the B-HOT panel and the custom Elements AMR-specific panel.

## MATERIALS AND METHODS

### Sample Collection

A total of 96 for-cause kidney transplant biopsies was included in this 2-center retrospective study between 2009 and 2019. Three groups were selected: AMR (n = 32), borderline (BL) or TCMR (BLorTCMR, n = 32), and no rejection (NoRejection, controls, n = 32). All AMR samples were diagnosed as active AMR, of which 14 also had a component of BLorTCMR, reflecting a common clinical transplant setting, whereas the BLorTCMR cohort did not show any sign of AMR. The focus has been on active AMR, as this is the most challenging diagnosis of AMR with the decisive Banff Lesion Scores g and ptc showing worse reproducibility than cg.^3^ Seventy biopsies were retrieved from the archives of the Department of Pathology, Erasmus MC, Rotterdam, The Netherlands, and 26 biopsies from the Institute of Pathology, University Hospital Cologne, Cologne, Germany. All biopsies were reassessed according to the Banff 2019 Update.^15^ Banff lesion scores specific for microvascular inflammation (g and ptc) of the AMR samples are presented in Table S1 (**SDC**, http://links.lww.com/TP/C588). Biopsies without evidence of rejection showed acute tubular damage and a few biopsies also showed changes not attributable to rejection, for example, arteriolosclerosis, glomerulosclerosis, and ischemic changes in the glomeruli. The diagnostic categories according to Banff 2019 Update^15^ of all biopsies are presented in a spreadsheet (Table 1).

**TABLE 1.** Sample characteristics

Characteristics	NoRejection (n = 32)	BLorTCMR (n = 30)	AMR (n = 32)
Department of Pathology			
Rotterdam, The Netherlands—n (%)	32 (100)	19 (63.3)	17 (53.1)
Cologne, Germany—n (%)	0 (0)	11 (36.7)	15 (46.9)
Banff classification			
Category 2 (antibody-mediated changes)—n (%)	0 (0)	0 (0)	32 (100)
Category 3 (suspicious [borderline] for acute T cell–mediated rejection)—n (%)	0 (0)	8 (26.7)	9 (28.1)
Category 4 (T cell–mediated rejection)—n (%)	0 (0)	22 (73.3)	5 (15.6)
aTCMR IA—aTCMR IB		3 (10.0)–1 (3.3)	1 (3.1)–0 (0)
aTCMR IIA—aTCMR IIB		15 (50.0)–2 (6.7)	3 (9.3)–1 (3.1)
caTCMR IA—caTCMR IB		0 (0)–1 (3.3)	0 (0)–0 (0)
caTCMR II		0 (0)	0 (0)

Two BLorTCMR samples from Erasmus MC, Rotterdam, The Netherlands, had no material in the FFPE blocks for mRNA analysis.

AMR, antibody-mediated rejection; aTCMR, acute T cell–mediated rejection; BLorTCMR, borderline or T cell–mediated rejection; caTCMR, chronic active T cell–mediated rejection; FFPE, formalin-fixed paraffin-embedded; NoRejection, no rejection.

### RNA Isolation

RNA isolation of all samples was performed at the Department of Pathology at the Erasmus MC, Rotterdam. Three consecutive 20-µm sections were obtained from each FFPE block for RNA isolation. The sections were immediately transferred to sterile microcentrifuge tubes and stored at room temperature. Microtome blades were replaced and between blocks the equipment was sterilized with RNase AWAY (Life Technologies, Carlsbad, CA). Xylene deparaffinization and RNA isolation were carried out according to the manufacturer’s instructions with the RNeasy FFPE Kit. RNA concentration and subsequently both quality and quantity were measured by both NanoDrop 2000 spectrophotometer (Thermo Fisher Scientific, Waltham, MA) and Bioanalyzer (Agilent, Santa Clara, CA).

### Molecular Panel Selection

The B-HOT panel includes 758 genes covering the most pertinent genes from the core pathways and processes related to host responses to rejection of transplanted tissue, tolerance, drug-induced toxicity, transplantation-associated viral infections (BK polyomavirus, cytomegalovirus, Epstein-Barr virus) plus 12 internal reference genes for quality control and normalization.^14^

The smaller Elements panel was based on AMR-associated transcripts published in the table of genes in the Banff 2017 update^11^ and consists of 90 genes plus 6 internal reference genes for quality control and normalization. It includes the AMR-specific panels by Halloran et al, Roufosse et al, Venner et al, and Adam et al.^5-8^ Three genes from the Venner panel were not included (KLF2, TRDV3, HYAL2) in the Elements panel because of the space constraints of the NanoString cards. Of the 90 genes included in the Elements panel, 4 (APOBEC3A, CCL3, PGM5, SDR16C5) are not part of the B-HOT panel. The complete list of genes included in the custom Elements panel is provided in Table S2 (**SDC**, http://links.lww.com/TP/C588).

### NanoString nCounter Assay, Data Normalization, and Analysis

The B-HOT panel and the Elements panel were used to analyze gene expression in 96 FFPE samples using the NanoString nCounter FLEX analysis system (NanoString Technologies). Quality control and normalization of raw gene expression counts were performed with nSolver Analysis Software Version 4.0 (NanoString Technologies). The geNorm algorithm was applied to analyze the stability of the reference genes.^16^ The 3 best reference genes for the B-HOT panel, and the Elements panel were used for normalization. Default parameters for quality control flagging were used for imaging (all plates were evaluated with 490 fields of view, binding density (0.1–2.25), positive control linearity (R^2^ value > 0.95), positive control limit of detection (0.5 fM positive control ≥2 SDs above the mean of the negative controls), technical variation (correction factor 0.1–2.0), and input RNA normalization (correction factor 0.1–8.0). Samples were excluded if technical and reference gene normalization factors were outside of the acceptance window, and the geometric mean of the reference gene candidates was under 30 counts. Samples with binding densities outside of the window between 0.1 and 2.25 signals per square micrometer were accepted if the positive control linearity R2 value was >0.95, and pass criteria for positive control and reference gene correction factors were fulfilled. Finally, the background threshold was determined by calculating the background of every sample as the average negative control probes plus 2 standard deviations and successively multiplying the highest background by 2. Probes were excluded from the analysis when their count was lower than the background threshold in at least 50% of the samples.

### Statistical Analysis

For the B-HOT panel and the Elements panel, we used R-based nSolver Advanced Analysis Software Version 4.0 (NanoString Technologies) to evaluate normalized mRNA expression values for gene expression analysis. The median expression for each probe was calculated for the 3 groups (AMR, BLorTCMR, and NoRejection). Log2 Fold Change was obtained using the previously calculated median value. A Wilcoxon test was performed to assess significance of differential gene expression in pairwise comparisons. Differential gene expression data of the panels are represented as volcano plots showing the top differentially regulated genes. The false discovery rate (FDR; Benjamini-Yekutieli) method was used to adjust the *P* values for multiple *t*-testing. A *P* value of below 0.05 was considered significant in 2-sided tests. Additionally, to explore possible gene expression patterns within the dataset, the data from the B-HOT panel and the Elements panel were subjected to unsupervised hierarchical clustering analysis (HCA) based on Ward’s minimum variance method^17^ and Canberra metric,^18^ the sum of scaled absolute distances between 2 points represented as n-dimensional vectors (n ≥ 1). For both clustering analyses, the Hopkins statistic (H, a measure of the probability that a given dataset is generated by uniform data distribution) has been calculated to assess cluster tendency,^19^ the correlation between the original pairwise distances and cophenetic distances has been calculated as a measure of how appropriately the produced dendrogram summarized the analyzed data,^20^ and the c-index has been used to calculate the ideal number of clusters.^21^ For each panel, least absolute shrinkage and selection operators (LASSO)^22^ was used with normalized data to select genes predicting AMR versus no AMR (combination of BLorTCMR and NoRejection). LASSO was selected as a methodology for the good performances shown by models built using genomic data^23^ and in the field of pathway analysis.^24^ Predictors displaying high correlation were excluded before applying LASSO. Performance of the extracted models (B-HOT model and Elements model) was measured by the area under the receiver-operating–characteristic (ROC) curve (AUC) on the B-HOT panel and Elements panel. Determined by 10-fold cross-validation, regularization parameters (λ) of 0.034 and 0.120 were used for the B-HOT model and the Elements model, respectively. Successively, data from the B-HOT panel analysis served as a test set for the Elements model. Genes with variance near zero were excluded from the model building process.

### Ethical Permission

The study was approved by the institutional review board of the Erasmus MC (Medical Ethical Review Board number MEC-2019-0307). Under Dutch state law, it is permitted to use left-over paraffin tissue for medical research. The biopsies from Cologne are covered by (#11-116). This research has been performed following the Declaration of Helsinki.^25^ All transplant procedures have been performed in accordance with the Declaration of Istanbul.^26^ No transplants from prisoners have been used.

## RESULTS

### Quality Control of RNA Isolation and NanoString nCounter Gene Expression Analysis

**FIGURE 1.**
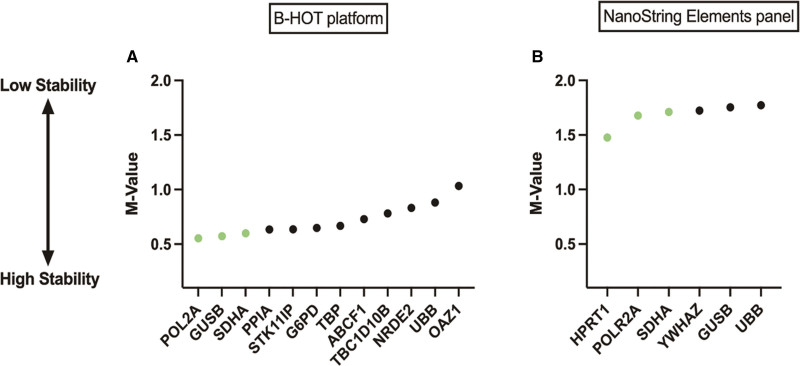
Reference genes stability value M by geNorm. B-HOT, Banff-human organ transplant.

NanoDrop assay showed a median RNA concentration of 29 ng/µL (1–179 ng/µL). Of the 96 biopsies, 2 BLorTCMR samples from the Erasmus MC had no material left in the FFPE blocks for mRNA isolation. One sample in the B-HOT panel and 8 samples in the Elements panel were excluded because of insufficient RNA. After mRNA isolation and NanoString nCounter run, 8 samples run with the B-HOT panel and 16 samples run with the Elements panel did not pass the quality control of the nSolver Advanced Analysis Software and were excluded from further analysis. Within the B-HOT panel analysis, 28 AMR samples, 26 BLorTCMR samples, and 31 NoRejection samples remained for further investigation. Within the Elements panel analysis, 25 AMR samples, 19 BLorTCMR samples, and 28 NoRejection samples remained for further investigation. With the geNorm algorithm, we identified the best reference genes for the B-HOT panel (Figure 1A) and the Elements panel (Figure 1B). The 3 most stable reference genes of the B-HOT panel were (with their respective stability value M in brackets) POLR2A (0.553), GUSB (0.573), and SDHA (0.600). The 3 most stable reference genes of the Elements panel were HPRT1 (1.477), POLR2A (1.677), and SDHA (1.711). Of the 770 probes in the B-HOT panel, 114 did not reach the detection threshold and of the 96 probes in the Elements panel, 13 did not reach the detection threshold. The data of the B-HOT panel and Elements panel analyses was normalized using their respective 3 most stable reference genes and then extracted from nSolver into R for the subsequent analysis.

### Retest Reliability

**FIGURE 2.**
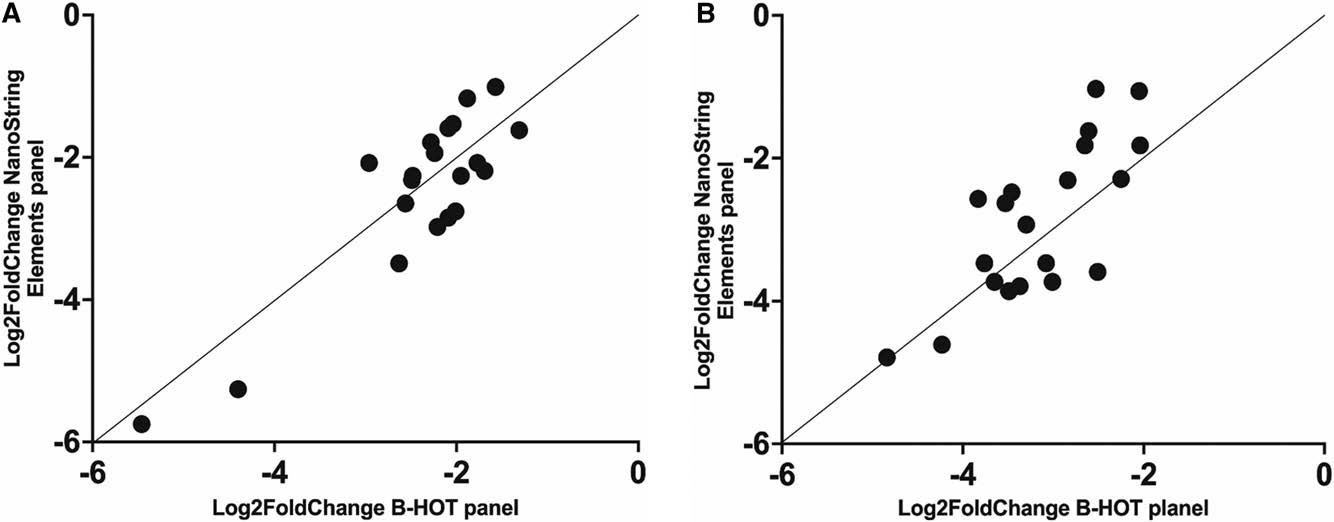
A, Correlation of overlapping genes of the B-HOT panel and the NanoString nCounter Elements panel comparing AMR versus no rejection. B, Correlation of overlapping genes of the B-HOT panel and the NanoString nCounter Elements panel comparing BLorTCMR versus no rejection. AMR, antibody-mediated rejection; B-HOT, Banff-human organ transplant; BLorTCMR, borderline or T cell–mediated rejection.

Of the 90 target genes in the Elements panel, 85 genes overlapped with the B-HOT panel and 5 genes did not (APOBEC3A, CCL3, PGM5, S1PR5, and SDR16C5). The 85 genes in both panels showed overlap in 57 target mRNA transcripts and no overlap in 28 target mRNA transcripts. Both panels contained 59 probes with the same probe ID and 31 probes with differing probe IDs. Log2-fold change of gene expression levels of the top 20 differentially expressed genes (DEGs) were highly correlated (Pearson’s coefficient: NR versus AMR = 0.88 (Figure 2A); NR versus TCMR = 0.76 (Figure 2B); TCMR versus AMR = 0.768) when comparing pairwise the same classes using the Elements panel and the B-HOT panel. Mean, SD, and coefficient of variation per diagnostic category for both panels are provided in Tables S3 and S4 (**SDC**, http://links.lww.com/TP/C588).

### Differential Gene Expression Using the B-HOT Panel

In an unsupervised HCA using the whole B-HOT panel probeset (**Figure S1, SDC**, http://links.lww.com/TP/C588), gene expression profiles of AMR, BLorTCMR, and NoRejection displayed a Hopkins statistics (H) >0.87, an optimal number of clusters of 3 (c-index = 0.249), and a correlation coefficient between pairwise cophenetic distances and original distances of 0.73.

#### NoRejection Versus BLorTCMR or AMR Using the B-HOT Panel

**FIGURE 3.**
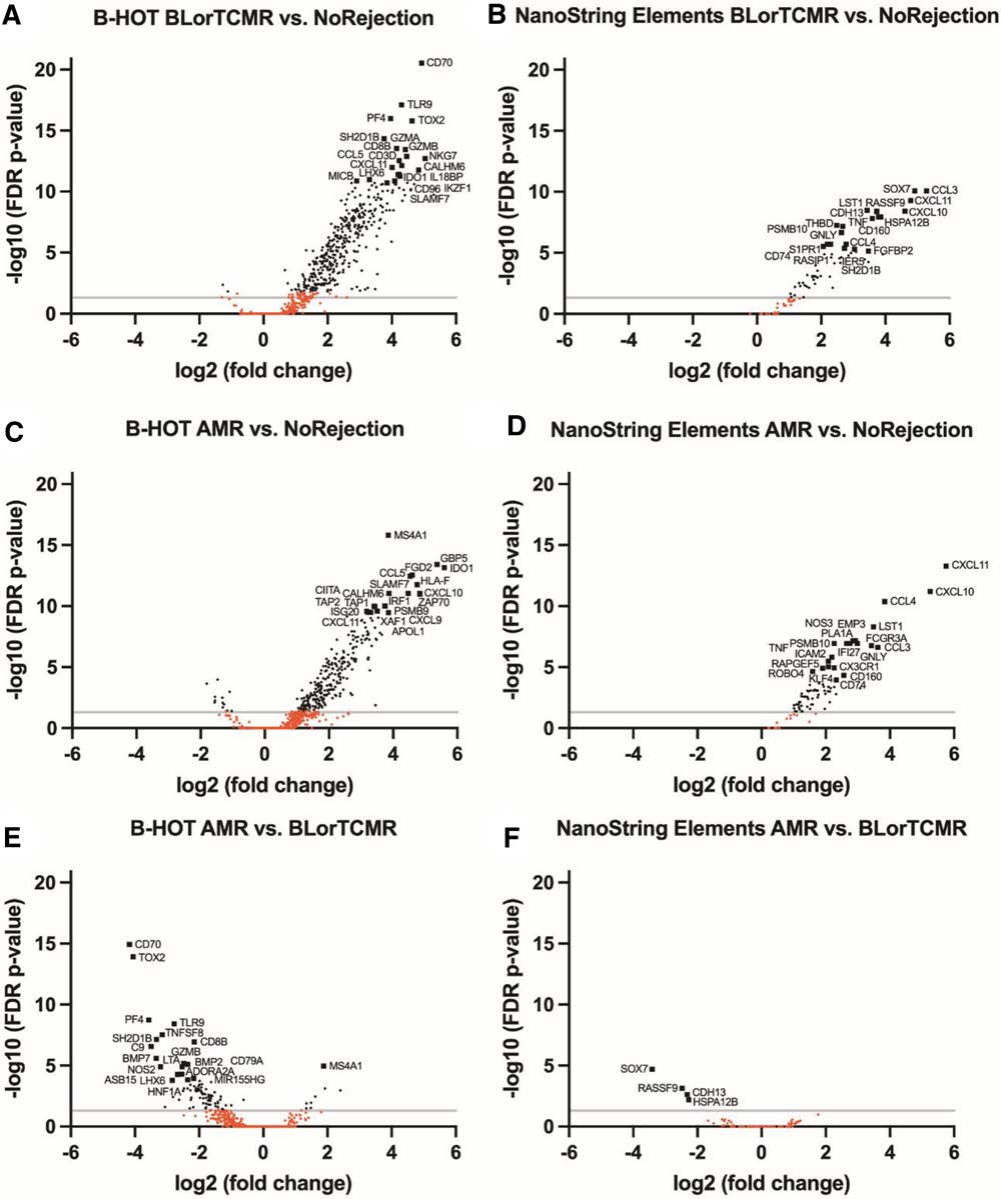
Volcano plots showing the pairwise comparison of the differential expression of genes between BLorTCMR, AMR, and NoRejection using the B-HOT panel and the NanoString nCounter Elements panel. AMR, antibody-mediated rejection; B-HOT, Banff-human organ transplant; BLorTCMR, borderline or T cell–mediated rejection; FDR, false discovery rate; NoRejection, no rejection.

Differential gene expression analysis in pairwise comparisons using the B-HOT panel identified a distinct gene expression pattern in both BLorTCMR and AMR biopsies compared with NoRejection biopsies (Figure 3A,C). Comparison of biopsies with BLorTCMR to those with NoRejection identified 353 genes with higher expression levels in the BLorTCMR samples (FDR *P* value [FDRPV] < 0.05–2.96e-21) and comparison of biopsies with AMR to those with NoRejection biopsies identified 294 genes with higher expression levels in the AMR samples (FDRPV < 0.05–1.57e-16). The top 20 DEGs per comparison are given in Table 2 and detailed information about these top 20 DEGs is displayed in Tables S5 and S6 (**SDC**, http://links.lww.com/TP/C588).

**TABLE 2.** Top 20 differentially expressed genes comparing samples with BLorTCMR, AMR, or NoRejection using the B-HOT panel

Ranking	BLorTCMR versus NoRejection (gene, FDRPV^a^)	AMR versus NoRejection (gene, FDRPV^a^)	AMR versus BLorTCMR (gene, FDRPV^a^)
**1**	CD70, 2.96e-21	MS4A1, 1.57e-16	CD70, 1.15e-15
**2**	TLR9, 7.82e-18	CXCL11, 3.79e-14	TOX2, 1.22e-14
**3**	PF4, 1.02e-16	GBP5, 7.13e-14	PF4, 1.84e-09
**4**	TOX2, 1.62e-16	SLAMF7, 2.85e-13	SH2D1B, 3.68e-09
**5**	SH2D1B, 4.59e-15	HLA-F, 3.57e-13	TLR9, 2.91e-08
**6**	GZMB, 2.91e-14	IDO1, 1.76e-12	TNFSF8, 7.03e-08
**7**	CD8B, 3.54e-14	CCL5, 9.23e-12	CD8B, 1.13e-07
**8**	NKG7, 1.28e-13	CXCL10, 9.29e-12	C9, 2.65e-07
**9**	IDO1, 1.92e-13	ZAP70, 9.29e-12	ADORA2A, 2.54e-06
**10**	CCL5, 2.88e-13	CXCL9, 1.1e-11	BMP2, 6.62e-06
**11**	CALHM6, 7.28e-13	ISG20, 9.72e-11	BMP7, 8.04e-06
**12**	GZMA, 1.04e-12	XAF1, 9.83e-11	MS4A1, 1.11e-05
**13**	CXCL11, 1.68e-12	IRF1, 9.83e-11	LTA, 1.21e-05
**14**	IKZF1, 3.8e-12	CALHM6, 9.93e-11	HNF1A, 1.23e-05
**15**	IL18BP, 4.14e-12	PSMB9, 1.1e-10	LHX6, 4.98e-05
**16**	SLAMF7, 5.46e-12	TAP2, 2.57e-10	NOS2, 5.37e-05
**17**	LHX6, 1.03e-11	CIITA, 2.62e-10	MIR155HG, 0.000104
**18**	CD3D, 1.32e-11	APOL1, 3.2e-10	GZMB, 0.000112
**19**	CD96, 1.32e-11	TAP1, 3.26e-10	ASB15, 0.000144
**20**	MICB, 1.89e-11	FGD2, 3.28e-10	CD79A, 0.000161

The top 20 DEG are in order of FDRPV, the gene with the lowest FDRPV is at the top.

^*a*^FDR *P* value was obtained from the adjusted *P* value of FDR correction by the Benjamini-Yekutieli method.

AMR, antibody-mediated rejection; BLorTCMR, borderline or T cell–mediated rejection; DEG, differentially expressed gene; FDR, false discovery rate; FDRPV, FDR *P* value; NoRejection, no rejection.

#### BLorTCMR Versus AMR Using the B-HOT Panel

Differential gene expression analysis in pairwise comparisons using the B-HOT panel identified a distinct gene expression pattern in BLorTCMR biopsies compared with AMR biopsies (Figure 3E). This comparison identified 90 genes with higher expression levels in the BLorTCMR samples (FDRPV < 0.05–1.15e-15) and 9 genes with higher expression levels in the AMR samples (FDRPV < 0.05–1.11e-05). The top 20 DEGs are given in Table 2 and detailed information about these top 20 DEGs is displayed in Table S7 (**SDC**, http://links.lww.com/TP/C588).

### Differential Gene Expression Using the Elements Panel

In an unsupervised HCA using the whole Elements panel probeset (**Figure S2, SDC**, http://links.lww.com/TP/C588), gene expression profiles of AMR, BLorTCMR, and NoRejection displayed a Hopkins statistics (H) >0.77, an optimal number of clusters of 5 (c-index = 0.232), and a correlation coefficient between pairwise cophenetic distances and original distances of 0.575.

#### NoRejection Versus BLorTCMR or AMR Using the Elements Panel

Differential gene expression analysis in pairwise comparisons using the Elements panel identified a distinct gene expression pattern in BLorTCMR or AMR biopsies compared with NoRejection biopsies (Figure 3B,D). Comparison of biopsies with BLorTCMR to those with NoRejection biopsies identified 57 genes with higher expression levels in the BLorTCMR samples (FDRPV < 0.05–8.41e-11) and a comparison of AMR to NoRejection biopsies identified 76 genes with higher expression levels in the AMR samples (FDRPV < 0.05–5.23e-14). The top 20 DEGs per comparison are given in Table 3 and detailed information about these top 20 DEGs is displayed in Tables S8 and S9 (**SDC**, http://links.lww.com/TP/C588).

**TABLE 3.** Top 20 differentially expressed genes comparing samples with BLorTCMR, AMR, or NoRejection using the NanoString nCounter Elements panel

Ranking	BLorTCMR versus NoRejection (gene, FDRPV^a^)	AMR versus NoRejection (gene, FDRPV^a^)	AMR versus BLorTCMR (gene, FDRPV^a^)
**1**	SOX7, 8.41e-11	CXCL11, 5.23e-14	SOX7, 2.04e-05
**2**	CCL3, 8.41e-11	CXCL10, 6.39e-12	RASSF9, 7.18 e-04
**3**	CXCL11, 5.11e-10	CCL4, 4.21e-11	CDH13, 2.35 e-03
**4**	TNF, 3.36e-09	LST1, 4.97e-09	HSPA12B, 6.42 e-03
**5**	RASSF9, 3.91e-09	EMP3, 6.8e-08	VWF, 0.101
**6**	CXCL10, 3.91e-09	NOS3, 6.8e-08	CD160, 0.250
**7**	LST1, 4.15e-09	PSMB10, 1.13e-07	ROBO4, 0.270
**8**	CD160, 1.14e-08	CD74, 1.13e-07	THBD, 0.270
**9**	HSPA12B, 1.15e-08	PLA1A, 1.13e-07	CCL3, 0.324
**10**	CDH13, 1.52e-08	IFI27, 1.13e-07	IER5, 0.324
**11**	PSMB10, 5.61e-08	FCGR3A, 1.71e-07	IFI27, 0.326
**12**	THBD, 6.76e-08	CCL3, 2.42e-07	TNF, 0.343
**13**	GNLY, 2.23e-07	CX3CR1, 1.53e-06	TEK, 0.385
**14**	CCL4, 2.05e-06	ICAM2, 3.29e-06	EMP3, 0.385
**15**	IER5, 2.05e-06	GNLY, 9.27e-06	CRIP2, 0.385
**16**	RASIP1, 2.05e-06	TNF, 1.13e-05	TRIB1, 0.452
**17**	S1PR1, 3.06e-06	RAPGEF5, 1.23e-05	RASIP1, 0.452
**18**	CD74, 4.34e-06	ROBO4, 2.25e-05	MEOX1, 0.525
**19**	FGFBP2, 5.01e-06	CD160, 4.58e-05	NOS3, 0.525
**20**	SH2D1B, 7.34e-06	KLF4, 1.14e-04	CCL4, 0.565

The top 20 DEG are in order of FDRPV, the gene with the lowest FDRPV is at the top.

^*a*^FDR PV was obtained from the adjusted *P* value of FDR correction by the Benjamini-Yekutieli method.

AMR, antibody-mediated rejection; BLorTCMR, borderline or T cell–mediated rejection; DEG, differentially expressed gene; FDR, false discovery rate; FDRPV, FDR *P* value; NoRejection, no rejection.

#### BLorTCMR Versus AMR Using the Elements Panel

Differential gene expression analysis in pairwise comparisons using the Elements panel identified a subtly different gene expression pattern in biopsies with BLorTCMR compared with AMR (Figure 3F). A comparison of BLorTCMR and AMR biopsies identified 4 genes with higher expression levels in the BLorTCMR samples (FDRPV < 0.05–2.04e-05) and no genes with higher expression levels in the AMR samples. The top 20 DEGs are given in Table 3 and detailed information about these top 20 DEGs is displayed in Table S10 (**SDC**, http://links.lww.com/TP/C588).

### Diagnostic Classifiers From B-HOT Panel and the Elements Panel

**FIGURE 4.**
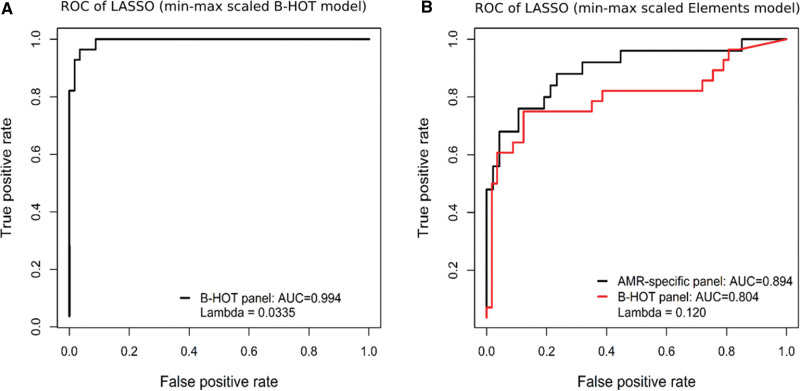
ROC curves demonstrating the diagnostic performance of the B-HOT and Elements models in discriminating AMR samples from non-AMR samples. AMR, antibody-mediated rejection; AUC, area under the curve; B-HOT, Banff-human organ transplant; Elements, NanoString nCounter Elements; LASSO, least absolute shrinkage and selection operator; ROC, receiver-operating characteristic.

Because of low variation, 107 genes were excluded before feature selection was performed on the normalized mRNA data from the B-HOT panel. Of the remaining genes from the B-HOT panel, LASSO analysis identified 24 genes associated with AMR that were included in the B-HOT model. The ROC curves and related AUCs of the B-HOT model, evaluated on the B-HOT panel data, are presented in Figure 4A. The B-HOT model achieved an AUC of 0.994 during cross-validation, with a sensitivity of 0.821 and a specificity of 1.00. A confusion matrix reporting on classification performance is presented in Table S11 (**SDC**, http://links.lww.com/TP/C588).

Because of low variation, 25 genes were excluded before feature selection was performed on the normalized mRNA data from the Elements AMR-specific custom panel. Of the remaining genes from the Elements panel, LASSO analysis identified 3 genes (CRIP2, EMP3, and PLAT) associated with AMR that were included in the Elements model. The ROC curves and related AUCs of the Elements model, evaluated on the Elements panel data, are presented in Figure 4B. The Elements model achieved an AUC of 0.894 during cross-validation, with a sensitivity of 0.480 and a specificity of 0.979. A confusion matrix reporting on classification performance is presented in Table S12 (**SDC**, http://links.lww.com/TP/C588).

When tested on data from the B-HOT panel analysis, the Elements model achieved an AUC of 0.804, with a sensitivity of 0.571 and a specificity of 0.965. The ROC curves and related AUCs of the Elements model, evaluated on the B-HOT panel data, are presented in Figure 4B. A confusion matrix reporting on classification performance is presented in Table S13 (**SDC**, http://links.lww.com/TP/C588).

## DISCUSSION

To the best of our knowledge, this pilot study is the first to investigate the feasibility of NanoString nCounter gene expression analysis using the recently introduced B-HOT panel to aid kidney transplant diagnosis and classification. In this study, we investigated technical aspects of this analysis such as the most stable reference transcripts for NanoString analysis of FFPE kidney transplant tissue and the reproducibility of the NanoString assay on kidney transplant biopsies in clinical practice. Successively, we investigated gene expression patterns of kidney transplant biopsies with different diagnoses (AMR, BLorTCMR, and NoRejection) and finally we explored the potential of these analyses for diagnosis and classification of rejection by developing 2 LASSO-based models to diagnose AMR, testing the one based on the smaller dataset (Elements model) on a dataset obtained from an analysis performed using a different panel (B-HOT panel).

The NanoString nCounter platform can use minimal tissue remaining on the FFPE block after standard histopathologic work-up. This is in great contrast with the Affymetrix assays used in the MMDx for which an additional fresh core needs to be obtained purely for transcriptomic analysis. In our hands, the panel fulfilled this promise, with adequate RNA yield in 88 of 96 blocks examined with the B-HOT panel and 80 of 96 blocks examined with the smaller Elements panel, even after splitting the recovered RNA into 2 aliquots for the 2 different assays. An important technical question concerning the NanoString technology is the choice of reference transcripts suited for kidney transplant tissue. We have found 4 gene transcripts (POLR2A, SDHA, GUSB, and HPRT1) to be the most stable, of which 2 genes (POLR2A and SDHA) were included in both the B-HOT panel and the Elements panel and 1 gene (GUSB) only in the B-HOT panel. HPRT1 was not included in the reference genes of the B-HOT panel.^5,7^

Our results confirm differential expression of transcript sets between biopsies without rejection versus BLorTCMR. As it stands, this approach of diagnosing Banff Category 3 or 4 might be of limited clinical value, only applicable after ruling out differential diagnoses like pyelonephritis or polyomavirus nephropathy. The use of molecular pathology could prove to be of additive diagnostic value to the Banff classification, with a potentially relevant role when differentiating between those cases of BLorTCMR that do need additional therapeutic intervention to halt transplant rejection. Clearly, more data from more centers and various control groups will be required to establish a molecular parameter and threshold for this diagnostic approach.

The diagnosis for which a molecular diagnostic tool is probably most eagerly awaited is AMR, with its remarkable histopathologic spectrum of different active and chronic lesions in the arteries, arterioles, glomeruli, peritubular capillaries, and—including C4d staining—medullary vasa recta. Several research groups are currently trying to use the NanoString nCounter platform and the B-HOT panel to fulfill the 2013 promise of providing a thoroughly validated diagnostic gene panel. Models developed filtering only the genes included in the B-HOT panel from the publicly available microarray datasets generated during the development of MMDx achieved performances that are comparable to those of MMDx during cross-validation.^27,28^ However, these studies did not assess performances of analog classifiers developed using data obtained with the NanoString nCounter platform, did not use the whole B-HOT panel because of few missing genes within the retrieved arrays, and did not attempt to validate the developed models to allow a robust comparison with MMDx. “Thorough validation,” reasonably involving multicentric prospective studies in large cohorts, is beyond the scope of this article, which is limited by its retrospective design, the relatively small number of cases, and the avoidance of cases that up until the 2017 Banff update^11^ were diagnosed as “suspicious for AMR.” However, we have provided promising pilot data using not only the NanoString nCounter platform and the B-HOT panel but also a customized AMR-specific Elements panel to support the role of molecular markers in aiding the difficult diagnosis of AMR. Beyond showing differential gene expression in an external retrospective bicentric test set, replicating a real-life diagnostic scenario of diagnosing AMR versus NoRejection, BLorTCMR, we were able to show an excellent AUC of 0.994, sensitivity of 0.821, and specificity of 1.00 for the B-HOT model during cross-validation and an AUC of 0.804, sensitivity of 0.571, and specificity of 0.965 for the Elements model when tested on data from the B-HOT panel analysis, a satisfying performance in relation to our sample size and to the exploratory nature of our model development attempt. From a clinical and practical point of view, being that this model is a model that should support an experienced nephropathologist, whereas analyzing ambiguous cases, it is crucial to achieve high specificities, also considering the low and difficult to estimate^29^ prevalence of comparable AMR cases. Our findings showcase the potential of this technology for this purpose. The use of extensive and high-quality datasets within the context of a multicentric study has the potential to achieve the excellent performances that are required for such a tool to be transferred into clinical practice.

Our results could indicate that smaller transcript sets of 75 genes (excluding reference transcripts) could suffice to aid in the classification of AMR with greater interobserver agreement than even experienced nephropathologists^3^ and that retest reliability seems sufficient between the full B-HOT and the smaller Elements panel. Indeed, our Elements model achieved satisfying performances on the B-HOT panel analysis test set (AUC = 0.804) only using 3 genes (CRIP2, EMP3, and PLAT) to classify the samples within the 3 different categories. However, we would still advocate the use of the entire B-HOT panel for now. Especially in a registry or research setting, it will be important to generate a multicentric, interchangeable database of not only common transcript sets but also shared assay numbers (probes) for these transcripts. Abandoning this consensus too soon would only lead to dead ends in research, slowing progress on this important topic. Furthermore, HCA of the B-HOT panel displayed a higher tendency to cluster (H > 0.87) and a better representation of the data (r = 0.73) when compared with HCA of the Elements panel (H > 0.77; r = 0.57), supporting its choice as the current reference gene panel.

Apart from that, several other issues remain. Even if the NanoString B-HOT panel should at some point be recognized as a “thoroughly validated” Banff Additional Diagnostic Parameter for the classification of AMR, at least in the current diagnostic framework, the AMR diagnosis will remain a diagnosis of exclusion (eg, with recurrent thrombotic microangiopathy in a setting of atypical hemolytic uremic syndrome). Moreover, the subtyping of AMR as active, chronic, or chronic active with its prognostic and therapeutic implications will require histomorphologic input from an experienced nephropathologist.

Practical and health-economic issues remain as well. Setting up, validating, and running a NanoString-based molecular diagnosis panel for the diagnosis of at least AMR requires considerable effort and will cost transplant centers at least $275 USD in consumables alone per test for the B-HOT panel (other consumables are not included in this quote).^14^ Although external service providers and the more widespread use of this panel might bring down the cost in the future, the currently high logistical and financial costs will rather discourage the implementation for all transplant biopsies in many transplant centers worldwide. The use of gene expression analysis will increase the cost of classifying kidney biopsy findings. However, in those cases that are not clear-cut, it could aid in superior classification and subsequent therapeutical choice. Ultimately this will lead to increased graft survival omitting the need for costly dialysis and improvement in quality of life of the transplant patient.

Nevertheless, the B-HOT panel is a considerable achievement of the Banff Foundation and the Molecular Diagnostics Working Group, deserving applause and support. To encourage and facilitate further research into the molecular diagnosis of AMR and beyond, we will provide the raw data of this study for future reference and meta-analyses, as is also the scope of the Banff Molecular Diagnostics Working Group.

In conclusion, the use of the B-HOT panel within the NanoString nCounter platform in a clinical setting to aid kidney transplant rejection classification proved to be feasible from a technical point of view and great potential seems to underlie the obtained gene expression patterns for the development of classification models. Additional research based on more extensive data collected in a multicentric setting is needed to build and validate a molecular tool that could be implemented with significant impact in clinical practice.

## Supplementary Material


