# Supplementary material for: Feasibility and Potential of Transcriptomic Analysis Using the NanoString nCounter Technology to Aid the Classification of Rejection in Kidney Transplant Biopsies
Source: Transplantation. 2022 Oct 27;107(4):903–12. doi: 10.1097/TP.0000000000004372 (PMC10065817; doi:10.1097/TP.0000000000004372)
Supplement: Supplementary file 1 [file tp-107-903-s001.pdf]

SUPPLEMENTAL DIGITAL CONTENT

Figures

Figure S1a. Heatmap of normalized gene expression values from the NanoString nCounter® B-HOT panel analysis.

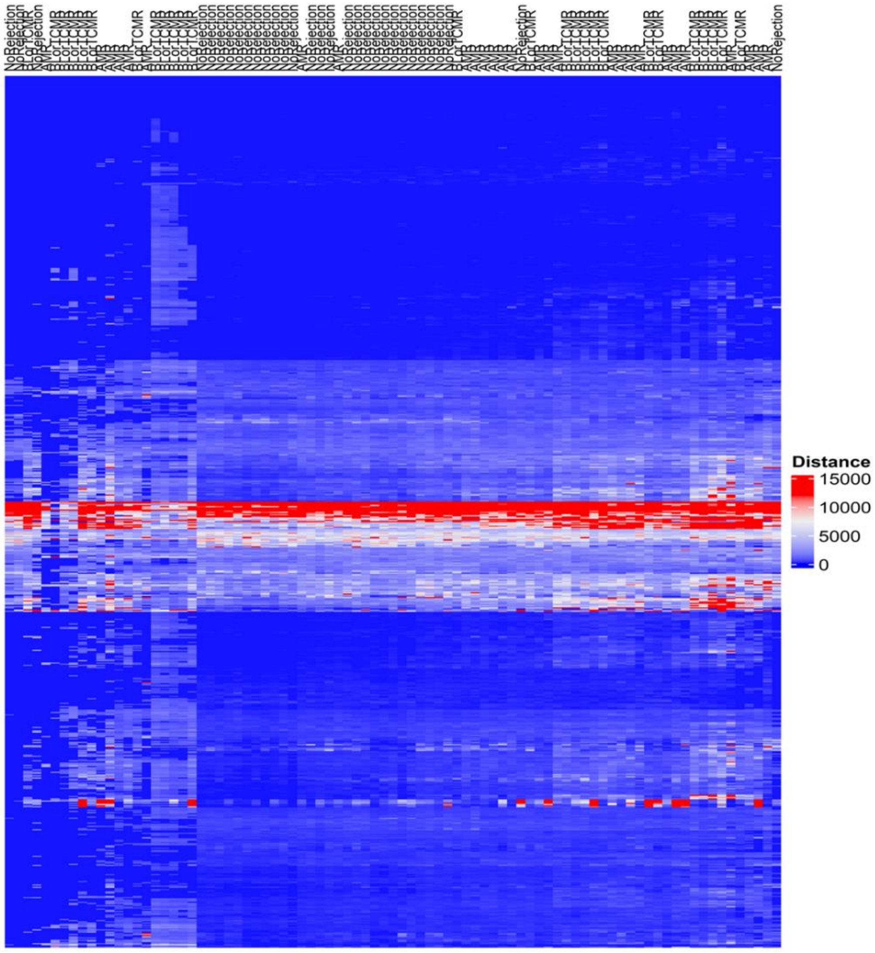

Figure S1a. Heatmap of normalized gene expression values from the NanoString nCounter® B-HOT panel analysis. Kidney transplant biopsies diagnosed with borderline or T cell-mediated rejection (BLorTCMR), antibody-mediated rejection (AMR), and no rejection (NoRejection).

Figure S1b. Dendrogram of unsupervised hierarchical cluster analysis of normalized gene expression values from the NanoString nCounter® B-HOT panel analysis.

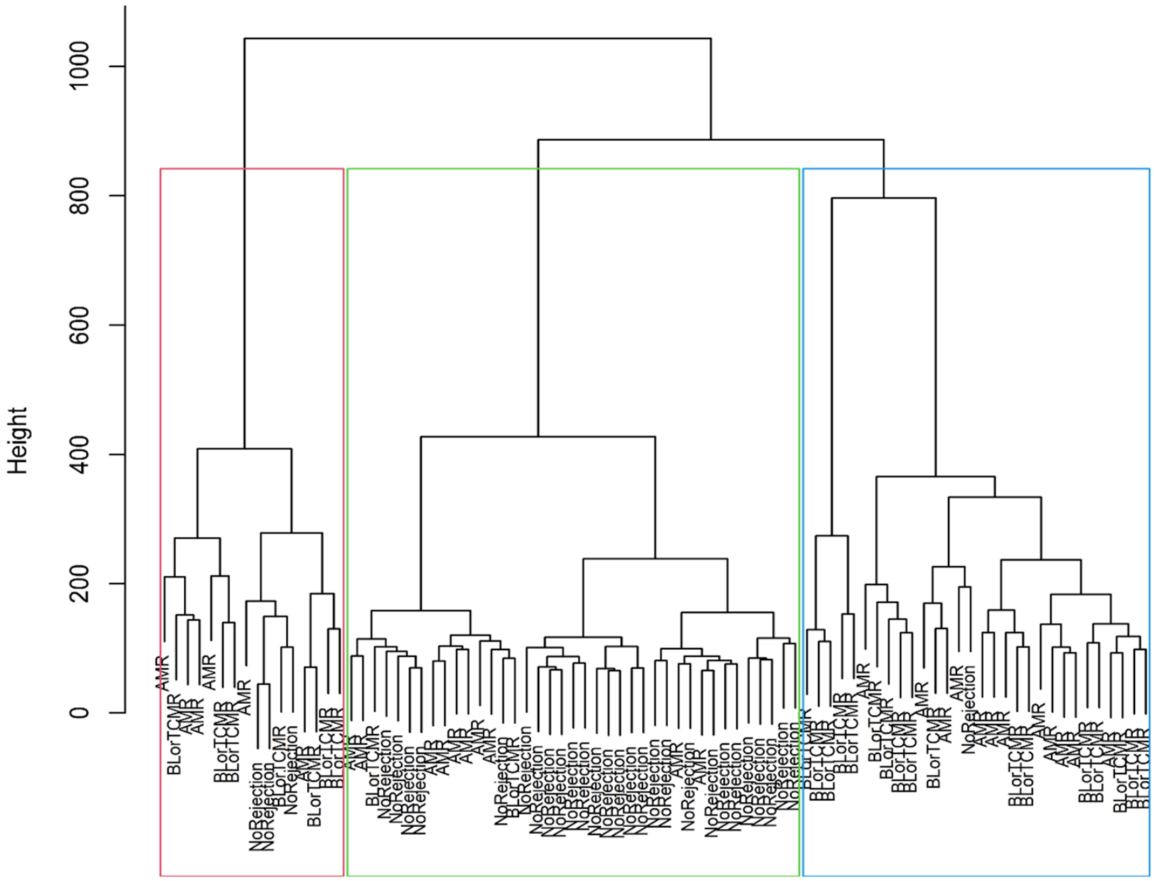

Figure S1b. Dendrogram of unsupervised hierarchical cluster analysis of normalized gene expression values from the NanoString nCounter® B-HOT panel analysis. Kidney transplant biopsies diagnosed with borderline or T cell-mediated rejection (BLorTCMR), antibody-mediated rejection (AMR), and no rejection (NoRejection).

Figure S2a. Heatmap of normalized gene expression values from the NanoString nCounter® Elements^{TM} AMR-specific custom panel analysis.

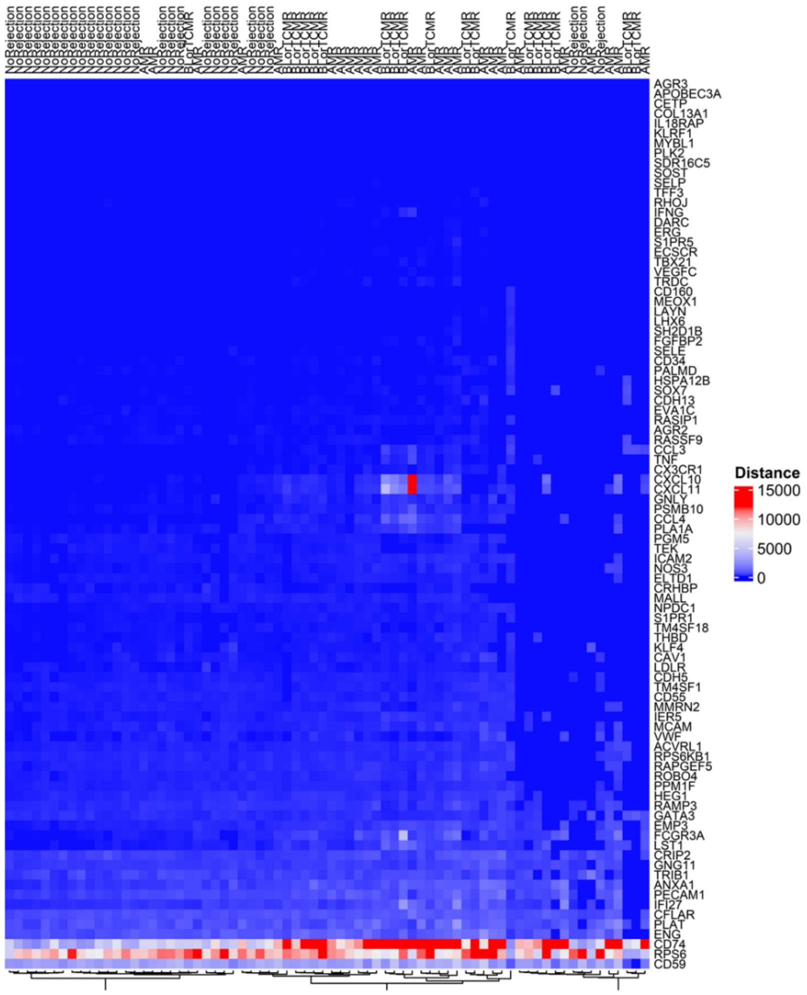

Figure S2a. Heatmap of normalized gene expression values from the NanoString nCounter® Elements^{TM} AMR-specific custom panel analysis. Kidney transplant biopsies diagnosed with borderline or T cell-mediated rejection (BLorTCMR), antibody-mediated rejection (AMR), and no rejection (NoRejection).

Figure S2b. Dendrogram of unsupervised hierarchical cluster analysis of normalized gene expression values from the NanoString nCounter® Elements™ AMR-specific custom panel analysis.

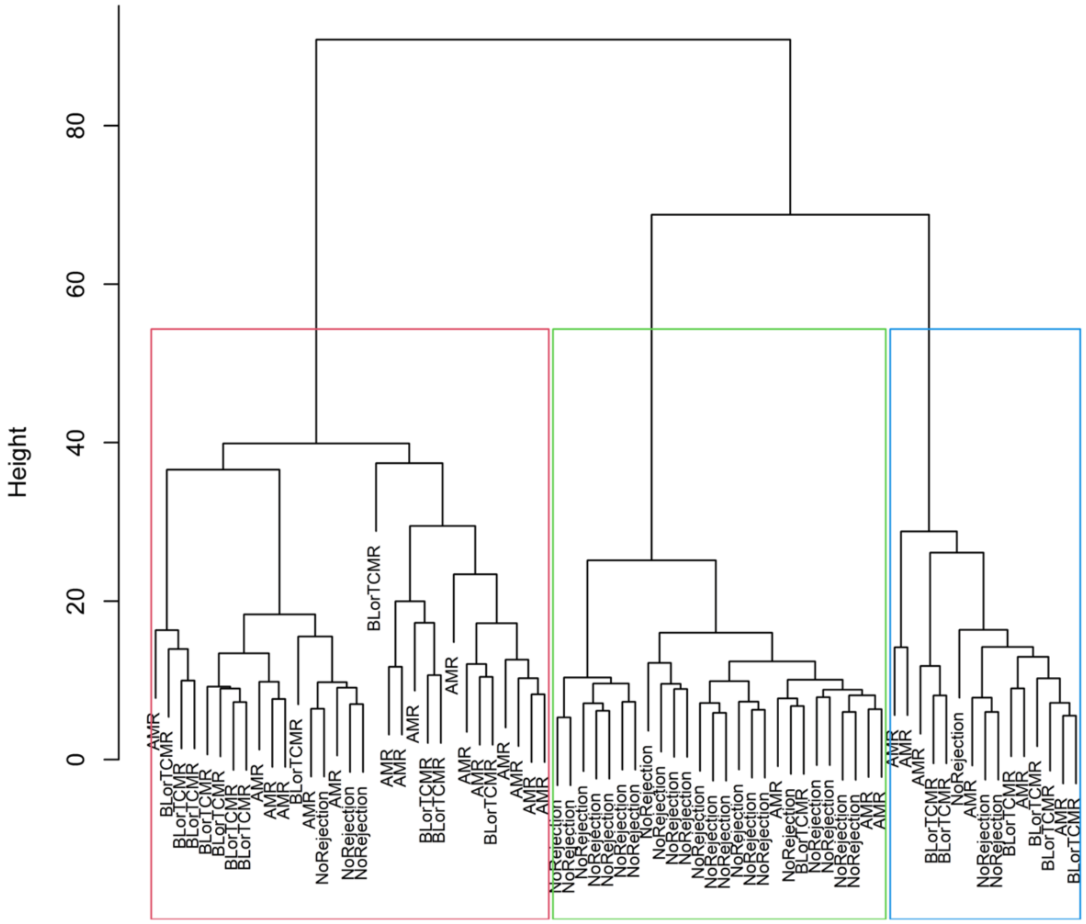

Figure S2b. Dendrogram of unsupervised hierarchical cluster analysis of normalized gene expression values from the NanoString nCounter® Elements™ AMR-specific custom panel analysis. Kidney transplant biopsies diagnosed with borderline or T cell-mediated rejection (BLorTCMR), antibody-mediated rejection (AMR), and no rejection (NoRejection).

Tables

Table 1S. Banff lesion scores specific for microvascular inflammation (g and ptc) of the samples classified with AMR. (g is glomerulitis and ptc is peritubular capillaritis)

Case	g	ptc	MVI
**1**	3	3	6
**2**	1	1	2
**3**	1	2	3
**4**	1	1	2
**5**	3	3	6
**6**	3	2	5
**7**	1	1	2
**8**	2	3	5
**9**	3	1	4
**10**	2	2	4
**11**	3	2	5
**12**	2	1	3
**13**	3	2	5
**14**	3	3	6
**15**	1	1	2
**16**	1	1	2
**17**	0	1	1
**18**	0	0	0
**19**	0	0	0
**20**	2	0	2
**21**	1	3	4
**22**	0	1	1
**23**	3	3	6
**24**	2	0	2
**25**	1	2	3
**26**	2	1	3
**27**	3	2	5
**28**	1	1	2
**29**	1	1	2
**30**	1	3	4
**31**	1	3	4
**32**	1	2	3

Table 2S. List of genes included in the NanoString nCounter® Elements™ AMR-specific custom panel.

ACVRL1	CRHBP	HSPA12B	NPDC1	S1PR5
AGR2	CRIP2	ICAM2	PALMD	SDR16C5
AGR3	CX3CR1	IER5	PECAM1	SELE
ANXA1	CXCL10	IFI27	PGM5	SELP
APOBEC3A	CXCL11	IFNG	PLA1A	SH2D1B
CAV1	DARC	IL18RAP	PLAT	SOST
CCL3	ECSCR	KLF4	PLK2	SOX7
CCL4	ELTD1	KLRF1	PPM1F	TBX21
CD160	EMP3	LAYN	PSMB10	TEK
CD34	ENG	LDLR	RAMP3	TFF3
CD55	ERG	LHX6	RAPGEF5	THBD
CD59	EVA1C	LST1	RASIP1	TM4SF1
CD74	FCGR3A	MALL	RASSF9	TM4SF18
CDH13	FGFBP2	MCAM	RHOJ	TNF
CDH5	GATA3	MEOX1	ROBO4	TRDC
CETP	GNG11	MMRN2	RPS6	TRIB1
CFLAR	GNLY	MYBL1	RPS6KB1	VEGFC
COL13A1	HEG1	NOS3	S1PR1	VWF

Table 3S. Overview of genes in the B-HOT panel with their corresponding mean, SD and CV in samples with AMR, NR or TCMR.

Gene	AMR			NR			TCMR		
	Mean	SD	CV	Mean	SD	CV	Mean	SD	CV
ABCA1	8,45	0,52	0,06	2,88	1,73	0,60	5,22	2,31	0,44
ABCB1	9,47	0,56	0,06	8,46	0,37	0,04	9,29	0,51	0,06
ABCC2	7,74	1,34	0,17	5,21	1,13	0,22	7,12	0,95	0,13
ABCE1	8,91	0,28	0,03	10,22	0,20	0,02	9,53	1,04	0,11
ACKR1	6,65	1,40	0,21	6,87	0,39	0,06	8,26	0,53	0,06
ACTA2	10,57	0,86	0,08	2,78	1,20	0,43	4,72	1,30	0,28
ACVR1	8,40	0,50	0,06	1,01	1,28	1,27	2,41	1,49	0,62
ACVRL1	9,01	0,44	0,05	5,01	0,80	0,16	5,92	1,71	0,29
ADAM8	5,08	0,99	0,20	8,33	1,02	0,12	9,71	1,44	0,15
ADAMDEC1	3,75	1,51	0,40	3,76	1,35	0,36	6,21	0,96	0,15
ADAMTS1	10,24	0,88	0,09	5,53	1,05	0,19	8,37	0,90	0,11
ADGRL4	8,37	0,50	0,06	4,98	1,07	0,22	7,48	1,10	0,15
ADORA2A	6,42	1,07	0,17	4,17	0,76	0,18	6,45	1,40	0,22
AGER	5,30	0,63	0,12	7,66	1,33	0,17	8,03	1,31	0,16
AGR2	6,53	0,72	0,11	7,91	1,68	0,21	8,94	0,99	0,11
AGR3	4,07	0,91	0,22	7,14	0,51	0,07	7,09	1,16	0,16
AGT	8,31	0,98	0,12	7,17	0,63	0,09	7,94	1,13	0,14
AHR	8,57	0,31	0,04	3,85	1,63	0,42	6,41	1,88	0,29
AICDA	2,83	2,29	0,81	7,51	1,22	0,16	8,12	2,38	0,29
AIM2	6,23	1,45	0,23	7,81	0,28	0,04	7,64	0,43	0,06
AIRE	3,77	1,79	0,48	6,09	0,67	0,11	6,68	0,83	0,12
AKR1C3	8,66	1,07	0,12	7,37	1,11	0,15	7,16	1,13	0,16
ALAS1	7,49	0,46	0,06	6,78	0,32	0,05	7,16	0,42	0,06
ALDH3A2	10,13	0,71	0,07	6,45	0,34	0,05	6,56	1,28	0,20
ALOX15	3,36	1,87	0,56	2,86	1,36	0,48	4,03	1,41	0,35
ALOX5	8,62	0,76	0,09	6,60	0,56	0,09	7,43	1,10	0,15
ANKRD1	5,28	2,03	0,38	7,72	0,73	0,10	8,08	0,74	0,09
ANKRD22	4,92	1,70	0,35	8,39	1,89	0,23	10,15	2,27	0,22
ANXA1	10,19	0,72	0,07	7,02	0,43	0,06	8,04	0,47	0,06
AOAH	7,50	0,79	0,10	7,83	0,39	0,05	8,29	0,87	0,10
APOE	12,22	1,18	0,10	2,48	1,64	0,66	5,03	2,71	0,54
APOL1	9,66	0,93	0,10	4,63	1,24	0,27	8,24	1,25	0,15
APOL2	8,74	0,85	0,10	3,82	0,95	0,25	5,10	1,17	0,23
APOLD1	6,48	1,20	0,18	2,97	1,23	0,41	5,80	1,41	0,24
AQP1	12,63	1,25	0,10	1,83	1,73	0,94	4,85	2,70	0,56
AQP2	8,18	1,55	0,19	8,58	0,54	0,06	8,68	0,54	0,06
AREG	3,70	1,55	0,42	9,52	0,16	0,02	8,70	1,21	0,14
ARG1	2,93	1,71	0,58	11,83	0,29	0,02	10,98	0,71	0,06
ARG2	8,91	0,87	0,10	8,88	0,31	0,03	9,07	0,68	0,07
ARHGDIB	10,94	0,67	0,06	9,84	0,34	0,03	9,26	1,14	0,12

ARRB2	7,42	0,76	0,10	6,78	1,00	0,15	9,39	0,87	0,09
ASB15	5,98	1,46	0,24	10,08	0,47	0,05	10,75	1,52	0,14
ATF3	7,61	1,58	0,21	7,13	1,03	0,14	8,54	0,64	0,08
ATM	8,64	0,38	0,04	4,00	0,88	0,22	6,15	1,77	0,29
ATXN3	8,29	0,28	0,03	5,86	1,13	0,19	8,21	0,77	0,09
AXL	7,83	0,69	0,09	8,13	0,64	0,08	8,85	0,54	0,06
B2M	14,72	0,99	0,07	4,76	1,26	0,27	7,59	1,00	0,13
B3GAT1	3,96	1,35	0,34	6,83	0,68	0,10	8,69	0,64	0,07
BASP1	7,94	0,87	0,11	10,02	0,17	0,02	10,27	0,27	0,03
BATF	5,31	0,96	0,18	8,73	0,96	0,11	9,72	0,85	0,09
BATF3	5,67	0,99	0,17	8,70	1,06	0,12	9,89	1,13	0,11
BAX	9,69	0,33	0,03	7,33	0,65	0,09	7,85	0,61	0,08
BCL2	7,62	0,60	0,08	8,36	0,27	0,03	8,33	0,51	0,06
BCL2A1	6,48	1,48	0,23	3,09	1,18	0,38	5,31	2,04	0,38
BCL2L1	9,30	0,38	0,04	2,99	1,65	0,55	5,42	2,56	0,47
BCL2L11	6,87	0,63	0,09	2,75	1,15	0,42	3,96	1,29	0,33
BCL3	8,32	0,78	0,09	3,99	1,22	0,31	6,79	1,49	0,22
BCL6	7,52	0,58	0,08	9,98	0,50	0,05	9,75	0,52	0,05
BDNF	3,56	1,70	0,48	9,27	0,41	0,04	9,71	0,58	0,06
BIRC3	6,87	1,13	0,16	3,81	0,63	0,16	4,61	1,05	0,23
BK,large,T,Ag	1,42	1,33	0,94	11,76	0,29	0,02	10,63	1,42	0,13
BK,,VP1	3,05	1,09	0,36	4,11	1,16	0,28	5,52	1,46	0,27
BLK	3,90	1,46	0,37	6,98	0,78	0,11	8,42	1,45	0,17
BLNK	7,49	0,47	0,06	3,94	1,60	0,41	6,50	1,98	0,31
BMP2	6,86	0,72	0,11	4,63	0,98	0,21	7,19	1,26	0,18
BMP4	6,95	0,97	0,14	2,45	1,78	0,73	4,85	2,66	0,55
BMP6	6,85	1,03	0,15	9,12	0,69	0,08	9,75	0,88	0,09
BMP7	6,53	0,84	0,13	4,64	1,00	0,21	6,13	1,28	0,21
BMPER	3,30	1,38	0,42	6,71	1,15	0,17	9,18	0,96	0,11
BMPR1A	8,78	0,61	0,07	8,51	0,54	0,06	8,47	0,53	0,06
BMPR1B	6,79	1,05	0,15	8,64	0,79	0,09	9,19	1,08	0,12
BRWD1	8,52	0,35	0,04	7,63	0,32	0,04	7,87	0,34	0,04
BST2	10,04	0,75	0,07	4,64	1,53	0,33	4,58	1,69	0,37
BTG2	8,94	1,02	0,11	8,83	0,62	0,07	7,34	1,59	0,22
BTK	6,34	1,03	0,16	6,01	1,10	0,18	8,25	0,61	0,07
BTLA	6,08	1,52	0,25	7,68	0,44	0,06	8,46	0,63	0,07
C1QA	9,79	1,05	0,11	5,33	1,04	0,20	7,13	1,56	0,22
C1QB	10,16	1,10	0,11	1,61	1,60	1,00	3,91	2,74	0,70
C1S	9,82	0,62	0,06	2,08	2,03	0,98	4,50	3,30	0,73
C3	8,47	1,55	0,18	6,60	0,56	0,09	6,44	0,74	0,11
C3AR1	7,42	0,76	0,10	9,50	0,23	0,02	9,33	0,63	0,07
C5	5,90	0,53	0,09	6,60	0,56	0,09	7,05	1,23	0,17
C5AR1	7,09	0,67	0,09	3,54	0,79	0,22	4,54	1,48	0,33
C9	5,20	1,29	0,25	7,14	0,89	0,12	8,02	0,56	0,07
CALHM6	8,70	1,36	0,16	9,92	0,35	0,03	9,92	0,42	0,04
CARD16	5,69	1,05	0,18	10,16	1,47	0,14	11,63	2,03	0,17

CARD8	8,05	0,52	0,06	7,23	0,74	0,10	8,98	0,66	0,07
CASP1	7,80	0,60	0,08	10,00	0,26	0,03	10,02	0,61	0,06
CASP3	8,07	0,35	0,04	2,80	1,38	0,49	5,05	1,78	0,35
CASP4	8,49	0,51	0,06	6,46	0,65	0,10	6,63	0,81	0,12
CASP8	8,55	0,41	0,05	4,25	1,08	0,25	6,80	0,80	0,12
CAV1	7,19	0,88	0,12	5,25	0,71	0,13	5,35	0,76	0,14
CCL13	5,50	1,08	0,20	6,53	0,36	0,06	6,75	0,59	0,09
CCL15	7,68	0,87	0,11	10,83	0,89	0,08	9,54	1,11	0,12
CCL18	6,31	1,77	0,28	7,65	0,86	0,11	8,85	1,62	0,18
CCL19	8,08	1,51	0,19	2,37	0,96	0,40	3,28	1,20	0,37
CCL2	9,57	1,10	0,12	10,40	1,07	0,10	10,61	1,63	0,15
CCL20	4,89	1,59	0,33	4,30	0,86	0,20	5,72	1,25	0,22
CCL21	8,07	1,39	0,17	3,21	1,47	0,46	5,96	2,29	0,38
CCL22	4,15	1,39	0,33	3,17	1,31	0,41	5,42	1,34	0,25
CCL3,L1	7,00	1,23	0,18	8,24	0,27	0,03	7,90	0,28	0,04
CCL4	4,13	1,78	0,43	4,70	0,84	0,18	5,75	0,99	0,17
CCL5	9,07	1,20	0,13	6,59	0,56	0,08	6,89	0,79	0,11
CCR1	7,11	1,04	0,15	3,46	1,44	0,42	6,01	1,82	0,30
CCR10	4,16	1,68	0,40	4,34	1,24	0,29	7,10	0,75	0,11
CCR2	7,13	1,03	0,14	6,44	0,35	0,05	7,03	1,24	0,18
CCR3	3,51	2,23	0,64	8,22	0,35	0,04	8,57	0,43	0,05
CCR4	4,95	1,73	0,35	12,87	0,43	0,03	12,18	1,29	0,11
CCR5	6,93	0,96	0,14	8,33	0,42	0,05	7,67	0,68	0,09
CCR6	5,19	1,40	0,27	7,59	0,43	0,06	8,86	0,46	0,05
CCR7	4,22	1,69	0,40	4,14	1,23	0,30	6,81	1,20	0,18
CD14	9,59	0,68	0,07	6,18	1,00	0,16	7,26	1,16	0,16
CD160	4,64	1,53	0,33	9,85	0,43	0,04	8,94	0,60	0,07
CD163	9,90	1,07	0,11	8,99	0,44	0,05	9,36	0,39	0,04
CD19	3,97	1,85	0,47	9,89	0,44	0,04	10,65	1,46	0,14
CD1D	5,90	0,83	0,14	4,49	1,09	0,24	6,00	1,05	0,18
CD2	6,02	1,03	0,17	8,47	0,92	0,11	9,82	1,35	0,14
CD207	3,41	1,28	0,38	7,10	0,50	0,07	7,22	0,79	0,11
CD209	7,43	1,03	0,14	7,60	0,31	0,04	7,22	1,01	0,14
CD22	5,17	2,17	0,42	3,22	1,61	0,50	5,00	2,23	0,45
CD24	13,10	0,79	0,06	7,85	0,44	0,06	7,73	0,49	0,06
CD244	4,35	1,34	0,31	8,75	0,32	0,04	7,81	1,10	0,14
CD247	7,21	1,05	0,15	8,20	0,31	0,04	8,35	0,60	0,07
CD27	6,08	2,24	0,37	8,18	0,18	0,02	8,45	0,45	0,05
CD274	5,83	1,36	0,23	9,13	0,39	0,04	9,26	2,01	0,22
CD276	8,05	0,48	0,06	6,98	0,78	0,11	8,89	0,75	0,08
CD28	6,18	1,21	0,20	9,07	0,48	0,05	9,22	1,33	0,14
CD34	7,08	0,90	0,13	2,66	1,59	0,60	5,14	2,18	0,42
CD38	7,06	1,34	0,19	2,94	1,90	0,65	5,70	2,70	0,47
CD3D	7,13	1,17	0,16	4,47	1,44	0,32	6,89	1,15	0,17
CD3E	6,87	1,14	0,17	9,43	0,36	0,04	9,10	0,44	0,05
CD3G	5,65	1,35	0,24	8,21	0,38	0,05	8,42	0,42	0,05

CD4	8,67	0,67	0,08	5,68	0,49	0,09	6,65	1,02	0,15
CD40	7,99	0,69	0,09	7,68	0,59	0,08	8,07	0,44	0,05
CD40LG	5,14	1,27	0,25	5,71	1,20	0,21	8,33	0,78	0,09
CD44	9,92	0,90	0,09	9,79	0,75	0,08	10,99	1,55	0,14
CD45R0	8,74	0,81	0,09	9,95	0,35	0,04	9,26	1,08	0,12
CD45RA	6,06	1,38	0,23	3,47	1,33	0,38	5,98	2,11	0,35
CD45RB	7,19	1,19	0,17	4,06	1,21	0,30	6,65	1,23	0,18
CD46	10,85	0,50	0,05	2,02	1,69	0,83	4,70	2,71	0,58
CD47	9,33	0,52	0,06	8,98	0,53	0,06	8,25	0,64	0,08
CD48	8,61	1,06	0,12	3,78	1,38	0,36	7,06	1,29	0,18
CD5	5,94	1,08	0,18	6,97	0,47	0,07	7,36	0,38	0,05
CD55	7,96	0,58	0,07	1,64	1,71	1,04	4,14	3,11	0,75
CD58	8,59	0,28	0,03	2,41	1,81	0,75	5,01	2,98	0,59
CD59	12,70	0,56	0,04	5,90	1,00	0,17	7,35	1,30	0,18
CD6	6,09	1,24	0,20	2,99	1,12	0,37	4,90	1,97	0,40
CD68	9,62	0,74	0,08	2,54	1,40	0,55	4,35	2,51	0,58
CD69	7,28	0,91	0,12	12,85	1,54	0,12	11,24	1,73	0,15
CD7	6,02	1,13	0,19	3,81	0,99	0,26	6,44	1,05	0,16
CD70	5,06	1,52	0,30	6,33	0,41	0,07	7,31	0,47	0,06
CD72	6,03	1,32	0,22	4,06	1,21	0,30	5,27	1,26	0,24
CD74	14,43	0,92	0,06	9,99	0,70	0,07	11,36	1,39	0,12
CD79A	4,95	2,19	0,44	6,51	1,14	0,18	9,10	1,45	0,16
CD80	3,50	3,65	1,04	7,68	0,35	0,05	7,28	0,73	0,10
CD81	12,49	0,64	0,05	0,56	2,65	4,69	5,42	2,91	0,54
CD82	7,65	0,69	0,09	4,51	1,13	0,25	7,46	1,26	0,17
CD83	8,27	0,82	0,10	4,40	1,01	0,23	6,60	0,83	0,13
CD84	8,22	0,71	0,09	6,82	0,66	0,10	5,25	1,02	0,19
CD86	6,15	1,01	0,16	1,62	1,35	0,83	3,71	2,91	0,78
CD8A	7,42	1,41	0,19	4,02	1,15	0,29	6,84	1,08	0,16
CD8B	5,77	1,42	0,25	4,39	1,29	0,29	7,15	0,89	0,12
CD96	6,83	1,03	0,15	7,48	0,66	0,09	8,55	0,79	0,09
CDH13	6,04	1,20	0,20	3,15	0,97	0,31	3,41	1,24	0,36
CDH5	7,61	0,59	0,08	4,71	1,04	0,22	7,34	2,24	0,31
CDKN1A	8,66	0,95	0,11	8,77	0,52	0,06	8,82	0,71	0,08
CEACAM3	3,29	1,87	0,57	7,23	1,38	0,19	9,23	0,98	0,11
CETP	4,99	0,89	0,18	2,78	2,38	0,85	3,80	2,66	0,70
CFB	9,62	1,05	0,11	7,16	0,53	0,07	7,88	0,75	0,10
CFH	9,06	0,71	0,08	4,54	0,70	0,15	5,68	1,19	0,21
CFI	10,28	0,91	0,09	8,41	0,46	0,05	8,05	0,65	0,08
CFLAR	9,69	0,27	0,03	8,25	1,19	0,14	9,76	1,28	0,13
CGAS	5,97	0,72	0,12	10,04	0,97	0,10	8,99	0,74	0,08
CH25H	3,75	1,67	0,45	6,47	1,46	0,23	9,27	2,04	0,22
CHCHD10	11,40	0,48	0,04	4,02	0,90	0,22	4,90	1,39	0,28
CHUK	8,29	0,25	0,03	8,71	0,55	0,06	9,78	0,53	0,05
CIITA	8,83	0,77	0,09	7,11	0,84	0,12	9,24	0,59	0,06
CITED4	6,59	0,79	0,12	6,92	1,21	0,18	9,38	0,81	0,09

CLEC4C	3,80	1,20	0,32	6,95	0,63	0,09	8,47	1,03	0,12
CMKLR1	8,51	0,63	0,07	6,29	1,15	0,18	8,70	0,96	0,11
CMV,UL83	2,22	1,04	0,47	7,23	0,62	0,09	7,33	0,57	0,08
COL13A1	4,80	0,90	0,19	3,80	1,40	0,37	6,52	1,68	0,26
COL1A1	9,84	1,35	0,14	2,77	1,17	0,42	4,26	1,15	0,27
COL3A1	12,38	1,11	0,09	2,48	1,85	0,75	5,09	2,63	0,52
COL4A1	11,38	0,82	0,07	3,29	1,03	0,31	5,16	0,97	0,19
COL4A3	7,44	0,45	0,06	11,54	0,38	0,03	11,64	0,41	0,04
COL4A4	9,49	0,38	0,04	8,79	0,69	0,08	9,59	0,80	0,08
COL4A5	6,79	0,81	0,12	3,24	1,21	0,37	6,40	1,71	0,27
CPA3	5,64	1,98	0,35	8,21	0,63	0,08	9,19	0,82	0,09
CR1	7,75	0,74	0,10	9,53	0,43	0,05	9,09	1,25	0,14
CRHBP	7,38	1,09	0,15	3,80	1,78	0,47	5,74	2,42	0,42
CRIP2	8,06	0,65	0,08	3,62	1,33	0,37	6,23	1,14	0,18
CRP	2,76	1,51	0,55	8,42	0,61	0,07	7,91	1,15	0,15
CSF1	7,30	0,67	0,09	5,47	0,93	0,17	7,78	0,94	0,12
CSF2	2,84	1,99	0,70	7,85	1,00	0,13	9,74	1,11	0,11
CSF2RB	6,07	1,37	0,23	5,01	1,03	0,21	7,51	0,90	0,12
CSF3	3,50	1,49	0,43	8,27	0,73	0,09	9,58	1,02	0,11
CSF3R	5,67	1,14	0,20	5,89	0,62	0,10	5,02	0,93	0,19
CTLA4	5,61	1,59	0,28	9,55	0,34	0,04	9,98	0,30	0,03
CTNNB1	10,98	0,45	0,04	5,09	0,99	0,19	7,73	0,77	0,10
CTSL	9,61	0,82	0,09	8,10	0,33	0,04	8,33	0,35	0,04
CTSS	10,79	1,04	0,10	3,11	0,96	0,31	4,51	1,52	0,34
CTSW	6,72	1,18	0,18	12,12	0,35	0,03	11,23	0,74	0,07
CX3CL1	8,18	0,72	0,09	8,89	0,44	0,05	9,76	0,61	0,06
CX3CR1	6,76	0,99	0,15	13,01	0,70	0,05	11,47	1,20	0,11
CXCL1,2	8,42	1,03	0,12	8,23	0,30	0,04	7,65	0,71	0,09
CXCL10	8,87	2,00	0,23	2,95	0,85	0,29	5,51	1,52	0,27
CXCL11	8,59	2,17	0,25	7,61	0,23	0,03	8,28	0,53	0,06
CXCL12	9,35	0,88	0,09	11,22	0,19	0,02	10,57	0,78	0,07
CXCL13	4,60	1,95	0,42	6,90	0,27	0,04	7,83	0,76	0,10
CXCL14	11,92	0,85	0,07	5,85	0,51	0,09	5,25	1,04	0,20
CXCL16	7,98	0,72	0,09	8,45	0,29	0,03	8,37	0,46	0,06
CXCL2	7,81	1,15	0,15	6,28	0,60	0,10	7,08	1,03	0,15
CXCL5	3,44	2,28	0,66	3,83	0,99	0,26	5,76	1,28	0,22
CXCL8	6,02	2,21	0,37	2,73	0,83	0,30	3,87	0,83	0,22
CXCL9	11,22	2,27	0,20	6,61	0,87	0,13	6,87	0,93	0,14
CXCR3	5,44	1,24	0,23	4,35	0,97	0,22	5,76	1,68	0,29
CXCR4	8,84	1,16	0,13	7,74	0,27	0,04	8,28	0,30	0,04
CXCR5	4,57	1,93	0,42	8,56	0,37	0,04	8,43	0,24	0,03
CXCR6	5,87	1,46	0,25	5,44	1,44	0,26	8,76	2,07	0,24
DCAF12	7,70	0,32	0,04	13,09	0,32	0,02	12,12	1,04	0,09
DDX50	8,38	0,51	0,06	2,87	1,12	0,39	4,99	2,17	0,44
DEFB1	10,23	0,88	0,09	8,98	0,27	0,03	9,04	0,27	0,03
DNMT1	7,96	0,33	0,04	5,58	0,95	0,17	7,27	1,15	0,16

DNMT3A	7,93	0,27	0,03	6,46	0,69	0,11	8,19	0,63	0,08
DUSP2	4,91	1,22	0,25	4,75	0,61	0,13	5,03	0,79	0,16
EBI3	5,33	1,16	0,22	2,04	1,02	0,50	2,54	0,82	0,32
EBV,LMP2	1,93	1,31	0,68	8,10	0,31	0,04	8,20	0,80	0,10
ECSCR	7,57	0,50	0,07	5,95	1,17	0,20	7,04	1,67	0,24
EDA	4,78	0,95	0,20	8,68	0,36	0,04	8,57	1,10	0,13
EEF1A1	15,31	0,44	0,03	7,72	0,32	0,04	7,93	0,64	0,08
EGFR	8,09	0,32	0,04	5,96	1,07	0,18	8,56	0,78	0,09
EGR1	7,58	1,47	0,19	6,87	0,37	0,05	7,11	1,05	0,15
EHD3	7,32	0,89	0,12	5,15	1,10	0,21	7,80	0,73	0,09
EMP3	8,29	0,70	0,08	9,09	0,49	0,05	9,23	0,57	0,06
ENG	10,18	0,72	0,07	5,32	1,08	0,20	7,82	0,76	0,10
EOMES	4,31	1,26	0,29	5,96	0,60	0,10	7,30	0,58	0,08
EPAS1	9,71	0,65	0,07	2,69	1,53	0,57	5,46	2,20	0,40
EPO	4,25	2,14	0,50	4,25	1,22	0,29	6,32	1,76	0,28
ERG	6,83	0,54	0,08	7,75	0,31	0,04	8,52	0,48	0,06
ERRFI1	9,29	1,23	0,13	8,52	0,21	0,03	8,58	0,49	0,06
EVA1C	6,81	0,49	0,07	8,20	0,48	0,06	9,03	1,26	0,14
EZH2	5,37	0,64	0,12	7,62	0,49	0,06	6,39	1,46	0,23
F3	6,66	0,86	0,13	4,37	0,75	0,17	6,20	1,21	0,20
FABP1	9,13	2,12	0,23	10,97	0,90	0,08	12,31	1,35	0,11
FADD	7,50	0,34	0,04	5,37	1,02	0,19	7,83	1,04	0,13
FAM30A	5,12	2,27	0,44	2,38	1,56	0,66	5,14	1,73	0,34
FAS	8,50	0,61	0,07	6,92	0,44	0,06	7,77	0,68	0,09
FASLG	4,87	1,15	0,24	6,02	0,59	0,10	7,06	0,98	0,14
FCAR	4,63	2,07	0,45	8,81	0,92	0,10	8,79	0,74	0,08
FCER1A	4,24	1,76	0,41	12,14	0,36	0,03	11,26	1,02	0,09
FCER1G	8,99	1,15	0,13	9,62	0,71	0,07	8,75	0,65	0,07
FCGR1A	7,51	1,23	0,16	8,74	0,35	0,04	8,28	0,66	0,08
FCGR2A	8,70	0,98	0,11	7,38	0,46	0,06	7,63	1,13	0,15
FCGR2B	7,25	1,26	0,17	7,07	0,39	0,06	7,27	0,66	0,09
FCGR3A,B	9,56	1,11	0,12	14,07	0,65	0,05	12,35	1,78	0,14
FCRL2	3,91	2,15	0,55	10,01	0,35	0,04	9,61	0,40	0,04
FGD2	8,76	0,99	0,11	7,38	1,15	0,16	7,19	0,84	0,12
FGFBP2	4,26	1,46	0,34	5,38	1,03	0,19	7,93	0,72	0,09
FJX1	6,63	0,92	0,14	3,07	1,58	0,52	5,29	2,14	0,40
FKBP1A	9,62	0,48	0,05	7,57	0,35	0,05	8,27	0,31	0,04
FLT3	5,71	0,93	0,16	7,56	0,44	0,06	8,19	0,56	0,07
FN1	10,22	1,26	0,12	6,82	1,04	0,15	7,70	1,18	0,15
FOS	10,16	1,80	0,18	11,95	0,49	0,04	10,10	1,79	0,18
FOSL1	5,37	1,44	0,27	6,57	0,81	0,12	7,59	1,33	0,18
FOXO1	8,31	0,46	0,06	3,93	1,14	0,29	6,20	1,37	0,22
FOXP3	3,61	1,85	0,51	3,04	1,01	0,33	4,34	1,23	0,28
FPR1	6,75	1,53	0,23	9,42	0,35	0,04	9,30	1,01	0,11
FYN	8,65	0,75	0,09	8,06	0,49	0,06	8,03	0,36	0,04
GAPDH	12,55	0,65	0,05	10,21	0,30	0,03	10,09	0,59	0,06

GATA3	9,01	0,66	0,07	6,74	0,56	0,08	7,09	1,23	0,17
GBP1	9,94	1,39	0,14	11,40	0,76	0,07	9,65	1,36	0,14
GBP2	9,17	1,02	0,11	3,82	1,43	0,37	5,95	1,99	0,34
GBP4	9,98	1,06	0,11	7,74	0,55	0,07	6,64	1,18	0,18
GBP5	7,77	1,91	0,25	11,78	0,52	0,04	12,07	0,74	0,06
GDF15	8,85	1,08	0,12	9,92	0,22	0,02	9,45	0,58	0,06
GEMIN7	7,42	0,81	0,11	9,84	0,68	0,07	10,02	1,07	0,11
GIMAP5	9,16	0,56	0,06	10,70	0,69	0,06	10,11	1,82	0,18
GNG11	9,10	0,49	0,05	8,56	0,23	0,03	8,35	0,27	0,03
GNLY	7,57	1,16	0,15	5,63	0,63	0,11	6,28	1,03	0,16
GZMA	7,96	0,91	0,11	7,48	0,93	0,12	8,45	0,75	0,09
GZMB	6,65	1,13	0,17	9,02	0,54	0,06	8,22	0,76	0,09
GZMH	5,99	1,36	0,23	2,84	1,22	0,43	5,63	1,42	0,25
GZMK	6,33	1,26	0,20	6,25	0,42	0,07	5,55	0,69	0,12
HAVCR1	8,08	1,52	0,19	5,84	1,56	0,27	8,23	2,49	0,30
HAVCR2	7,86	0,61	0,08	9,69	0,21	0,02	10,36	0,91	0,09
HDAC3	8,55	0,23	0,03	4,03	0,73	0,18	6,01	1,19	0,20
HDAC6	8,21	0,57	0,07	5,26	0,91	0,17	6,33	1,59	0,25
HDC	4,47	1,68	0,38	5,00	1,42	0,28	7,08	1,60	0,23
HEG1	9,27	0,47	0,05	4,02	0,52	0,13	6,48	1,20	0,19
HFE	6,03	0,75	0,12	4,49	0,94	0,21	6,12	0,73	0,12
HIF1A	10,84	0,65	0,06	6,17	1,11	0,18	8,60	0,93	0,11
HK2	6,46	1,53	0,24	9,26	0,50	0,05	9,05	0,64	0,07
HLA,A	14,15	0,82	0,06	8,39	0,57	0,07	8,43	0,82	0,10
HLA,B	12,63	0,96	0,08	13,99	0,52	0,04	14,03	2,03	0,14
HLA,C	12,81	0,86	0,07	8,64	0,34	0,04	9,28	1,00	0,11
HLA,DMA	10,02	0,80	0,08	5,62	0,70	0,13	6,88	1,06	0,15
HLA,DMB	8,84	0,61	0,07	2,03	1,24	0,61	2,69	1,12	0,42
HLA,DPA1	11,62	0,92	0,08	6,78	0,50	0,07	5,84	0,58	0,10
HLA,DPB1	11,30	0,90	0,08	3,20	1,03	0,32	5,68	1,75	0,31
HLA,DQA1	9,18	2,88	0,31	9,10	0,39	0,04	8,96	0,40	0,04
HLA,DQB1	10,10	1,07	0,11	6,63	0,74	0,11	7,71	1,21	0,16
HLA,DRA	13,25	0,93	0,07	4,69	0,78	0,17	4,80	1,13	0,24
HLA,DRB1	12,42	1,18	0,09	9,02	0,48	0,05	9,67	1,55	0,16
HLA,DRB3	12,23	0,93	0,08	5,19	0,85	0,16	6,57	1,85	0,28
HLA,E	12,15	0,73	0,06	7,51	0,72	0,10	8,98	0,66	0,07
HLA,F	8,82	0,95	0,11	2,72	1,02	0,37	5,43	1,65	0,30
HLA,G	5,81	0,99	0,17	7,80	0,24	0,03	6,80	1,01	0,15
HMGB1	9,90	0,49	0,05	11,35	0,34	0,03	11,55	1,50	0,13
HNF1A	6,53	0,93	0,14	6,49	0,99	0,15	8,84	0,56	0,06
HPRT1	8,59	0,42	0,05	2,94	1,67	0,57	5,23	2,25	0,43
HSD11B1	4,98	1,05	0,21	6,32	0,41	0,06	7,15	1,13	0,16
HSP90AA1	11,88	0,62	0,05	11,27	1,11	0,10	9,39	1,19	0,13
HSPA12B	7,07	0,91	0,13	7,09	0,47	0,07	8,51	1,22	0,14
HYAL1	8,96	0,67	0,08	8,36	0,52	0,06	7,71	1,10	0,14
HYAL2	9,67	0,52	0,05	3,21	1,35	0,42	5,35	1,60	0,30

ICAM1	8,80	1,12	0,13	6,80	0,30	0,04	7,46	0,39	0,05
ICAM2	7,81	0,61	0,08	7,17	0,88	0,12	9,16	0,55	0,06
ICOS	4,15	1,29	0,31	3,37	0,99	0,29	5,12	2,11	0,41
ICOSLG	6,84	0,45	0,07	6,84	0,61	0,09	7,61	0,47	0,06
IDO1	8,72	1,94	0,22	7,52	0,45	0,06	8,54	0,40	0,05
IER5	7,06	0,78	0,11	2,76	1,53	0,55	5,13	1,81	0,35
IFI27	10,02	0,82	0,08	11,85	0,66	0,06	12,73	1,72	0,14
IFI30	9,35	0,99	0,11	9,51	0,27	0,03	9,46	0,38	0,04
IFI44	8,48	0,91	0,11	10,78	0,53	0,05	10,72	0,68	0,06
IFI6	10,03	1,52	0,15	6,18	1,23	0,20	8,85	1,26	0,14
IFIT1	8,22	1,06	0,13	7,42	0,62	0,08	7,47	1,47	0,20
IFITM1	11,46	0,75	0,07	5,22	0,96	0,18	7,48	1,12	0,15
IFITM2	10,76	0,54	0,05	5,25	0,68	0,13	7,07	0,71	0,10
IFITM3	12,57	0,70	0,06	4,58	0,70	0,15	5,77	1,54	0,27
IFNA1	2,70	2,45	0,91	7,76	0,47	0,06	7,63	0,84	0,11
IFNAR1	9,08	0,74	0,08	9,64	0,47	0,05	8,79	1,35	0,15
IFNAR2	9,31	0,53	0,06	1,73	2,57	1,48	5,23	2,75	0,53
IFNG	4,21	1,56	0,37	7,10	0,55	0,08	7,66	0,54	0,07
IFNGR1	9,73	0,42	0,04	8,10	0,46	0,06	9,05	0,50	0,06
IFNGR2	9,40	0,64	0,07	5,60	0,84	0,15	7,01	0,87	0,12
IGF1	6,13	1,32	0,21	4,08	0,93	0,23	6,10	1,13	0,19
IGF1R	8,37	0,32	0,04	4,75	1,35	0,28	7,42	1,09	0,15
IGF2R	8,57	0,52	0,06	7,99	0,56	0,07	9,73	0,92	0,09
IGFBP1	3,61	1,26	0,35	3,27	0,88	0,27	4,15	1,50	0,36
IGFL1	1,30	1,61	1,24	3,45	1,44	0,42	5,50	2,44	0,44
IGHA1	8,56	3,26	0,38	7,17	0,38	0,05	7,72	0,65	0,08
IGHG1	10,09	3,93	0,39	2,38	1,10	0,46	3,57	0,87	0,24
IGHG2	10,52	3,67	0,35	4,62	1,10	0,24	7,16	1,49	0,21
IGHG3	10,59	3,65	0,34	10,58	0,83	0,08	9,27	1,03	0,11
IGHG4	9,43	3,63	0,39	2,74	1,35	0,49	4,67	2,79	0,60
IGHM	8,45	2,29	0,27	7,51	0,26	0,03	7,76	0,55	0,07
IGKC	10,97	3,51	0,32	9,05	0,38	0,04	8,89	0,53	0,06
IGLC1	11,60	2,59	0,22	2,12	2,42	1,14	5,68	2,29	0,40
IKBKB	8,20	0,45	0,05	7,13	0,69	0,10	6,54	0,94	0,14
IKBKG	7,60	0,54	0,07	5,70	0,65	0,11	6,47	1,13	0,17
IKZF1	8,06	0,95	0,12	5,03	1,36	0,27	6,90	1,40	0,20
IKZF2	6,53	0,74	0,11	1,28	0,91	0,71	1,71	1,50	0,88
IL10	3,96	0,88	0,22	7,95	2,01	0,25	7,93	2,22	0,28
IL10RA	8,51	0,74	0,09	6,52	0,30	0,05	6,90	0,50	0,07
IL10RB	7,56	0,80	0,11	12,80	0,69	0,05	12,08	1,28	0,11
IL12A	3,73	0,97	0,26	4,66	0,84	0,18	6,53	0,85	0,13
IL12B	3,24	2,02	0,62	8,07	0,51	0,06	9,17	0,55	0,06
IL12RB1	5,55	1,19	0,21	7,30	0,54	0,07	8,00	0,59	0,07
IL12RB2	4,66	1,17	0,25	6,94	0,89	0,13	8,93	0,48	0,05
IL13	2,88	1,97	0,68	5,75	0,87	0,15	7,49	1,35	0,18
IL15	7,11	0,64	0,09	6,86	0,64	0,09	7,20	1,24	0,17

IL16	7,72	0,99	0,13	5,68	0,84	0,15	8,00	0,69	0,09
IL17A	2,47	1,02	0,41	8,94	0,81	0,09	10,47	1,44	0,14
IL17F	2,60	2,46	0,95	3,92	0,73	0,19	4,74	0,78	0,17
IL17RA	7,94	0,54	0,07	7,89	0,18	0,02	7,68	0,26	0,03
IL17RB	9,77	1,45	0,15	7,15	0,32	0,04	6,57	0,88	0,13
IL17RC	7,41	0,28	0,04	8,97	0,22	0,02	9,01	0,35	0,04
IL18	8,32	0,53	0,06	8,08	0,51	0,06	8,49	0,60	0,07
IL18BP	8,07	1,16	0,14	7,42	0,52	0,07	8,67	0,72	0,08
IL18RAP	3,99	0,79	0,20	1,46	1,08	0,74	1,90	1,23	0,65
IL1A	2,67	2,42	0,91	4,09	1,75	0,43	7,16	1,06	0,15
IL1B	5,93	1,51	0,25	6,77	0,42	0,06	7,55	0,76	0,10
IL1R1	8,34	0,75	0,09	5,86	0,80	0,14	7,54	0,71	0,09
IL1R2	6,27	1,33	0,21	8,97	0,96	0,11	8,82	0,74	0,08
IL1RAP	5,92	0,96	0,16	5,97	0,62	0,10	7,06	0,89	0,13
IL1RL1	7,52	1,01	0,13	8,75	0,18	0,02	8,30	0,80	0,10
IL1RN	4,94	1,71	0,35	10,62	0,26	0,02	10,29	0,86	0,08
IL2	2,55	2,61	1,02	7,01	0,69	0,10	8,16	0,38	0,05
IL21	3,30	2,10	0,63	6,60	0,56	0,08	7,32	1,30	0,18
IL21R	5,76	1,04	0,18	10,25	0,36	0,04	9,79	0,67	0,07
IL22	2,18	2,36	1,08	2,55	1,72	0,67	5,09	2,26	0,44
IL23A	3,54	1,62	0,46	2,47	1,36	0,55	4,35	1,33	0,31
IL23R	3,78	1,77	0,47	7,29	0,95	0,13	7,06	1,41	0,20
IL27	3,71	2,24	0,60	6,89	0,46	0,07	7,85	0,58	0,07
IL27RA	6,91	0,73	0,11	7,43	0,38	0,05	7,71	0,52	0,07
IL2RA	5,82	1,19	0,20	7,74	0,62	0,08	8,14	0,50	0,06
IL2RB	7,28	1,03	0,14	9,67	1,63	0,17	9,64	1,37	0,14
IL2RG	7,91	0,98	0,12	5,33	0,82	0,15	6,50	1,42	0,22
IL33	6,89	0,72	0,10	3,46	1,34	0,39	6,45	1,07	0,17
IL4	2,54	2,27	0,89	2,74	1,32	0,48	4,62	0,99	0,21
IL4R	8,30	0,57	0,07	8,76	0,37	0,04	8,82	0,34	0,04
IL5	1,87	2,45	1,31	6,60	0,66	0,10	8,18	0,67	0,08
IL6	4,50	1,80	0,40	7,13	0,95	0,13	8,84	0,69	0,08
IL6R	7,90	0,41	0,05	7,62	0,32	0,04	7,39	0,67	0,09
IL6ST	11,35	0,30	0,03	6,99	0,61	0,09	8,44	0,67	0,08
IL7	4,35	1,30	0,30	8,93	0,68	0,08	10,23	0,71	0,07
IL7R	8,58	1,19	0,14	7,39	0,51	0,07	8,90	0,65	0,07
IMPDH1	7,91	0,73	0,09	8,13	0,21	0,03	8,43	0,41	0,05
IMPDH2	8,56	0,51	0,06	6,51	0,58	0,09	6,79	0,95	0,14
INHBC	3,70	1,91	0,52	10,53	0,74	0,07	10,14	0,90	0,09
INPP5D	8,22	0,64	0,08	3,11	1,40	0,45	5,29	2,40	0,45
IRF1	8,59	1,06	0,12	4,87	1,05	0,21	6,47	1,34	0,21
IRF4	6,52	1,81	0,28	5,54	0,71	0,13	7,49	0,57	0,08
IRF6	7,51	0,40	0,05	4,24	1,34	0,32	6,49	1,40	0,22
IRF7	7,16	1,07	0,15	8,53	0,91	0,11	8,11	1,12	0,14
IRF8	7,98	0,72	0,09	7,99	0,27	0,03	8,83	0,31	0,04
IRS1	7,33	0,63	0,09	7,48	0,56	0,08	9,08	0,98	0,11

ISG15	7,25	1,50	0,21	4,96	1,89	0,38	7,96	1,55	0,20
ISG20	7,76	1,30	0,17	10,31	0,20	0,02	10,35	0,93	0,09
ITGA4	7,47	0,87	0,12	7,87	1,45	0,18	11,07	2,31	0,21
ITGAM	6,12	0,92	0,15	4,24	1,22	0,29	6,22	1,90	0,31
ITGAX	7,45	1,13	0,15	8,68	0,25	0,03	8,97	0,32	0,04
ITGB2	8,29	1,02	0,12	7,74	0,73	0,09	7,63	0,85	0,11
ITGB6	9,07	1,17	0,13	7,27	1,36	0,19	8,25	0,79	0,10
JAK1	10,15	0,31	0,03	7,06	2,23	0,32	7,60	3,21	0,42
JAK2	8,35	0,45	0,05	3,88	0,66	0,17	5,13	0,71	0,14
JAK3	7,46	0,85	0,11	6,59	1,08	0,16	8,70	0,81	0,09
JUN	8,58	1,05	0,12	4,77	1,14	0,24	7,45	1,17	0,16
KAAG1	2,02	2,39	1,18	6,11	0,72	0,12	6,89	0,92	0,13
KDR	8,42	0,42	0,05	9,40	0,47	0,05	9,54	0,64	0,07
KIR_Activating_Subgroup_1	3,58	1,57	0,44	5,71	1,13	0,20	8,23	1,02	0,12
KIR_Activating_Subgroup_2	3,88	1,89	0,49	6,64	1,58	0,24	8,14	1,16	0,14
KIR_Inhibiting_Subgroup_1	3,76	0,90	0,24	1,78	1,37	0,77	3,24	0,94	0,29
KIR_Inhibiting_Subgroup_2	3,79	1,02	0,27	6,48	1,34	0,21	7,27	0,97	0,13
KIR3DL1	6,11	1,71	0,28	7,86	0,31	0,04	8,61	0,67	0,08
KIR3DL2	4,08	1,76	0,43	4,89	1,02	0,21	7,25	0,86	0,12
KIT	6,41	0,65	0,10	5,05	0,74	0,15	6,51	1,58	0,24
KITLG	9,07	0,36	0,04	9,29	0,32	0,03	8,96	0,56	0,06
KLF2	8,64	0,84	0,10	3,41	1,31	0,38	5,97	1,88	0,31
KLF4	7,84	0,79	0,10	10,03	0,28	0,03	9,74	0,76	0,08
KLHL13	8,02	0,56	0,07	2,82	1,33	0,47	4,39	2,09	0,47
KLRB1	7,12	1,12	0,16	9,50	0,85	0,09	8,71	0,72	0,08
KLRC1	4,95	1,34	0,27	5,96	0,79	0,13	5,77	0,82	0,14
KLRD1	4,50	1,49	0,33	8,53	0,78	0,09	9,07	1,40	0,15
KLRF1	4,18	0,91	0,22	6,03	1,23	0,20	9,36	1,51	0,16
KLRG1	5,82	1,00	0,17	5,10	1,42	0,28	6,68	1,51	0,23
KLRK1	7,34	1,07	0,15	7,45	0,85	0,11	8,93	1,45	0,16
KRT19	8,60	0,75	0,09	12,60	1,69	0,13	13,31	1,31	0,10
KRT8	11,59	0,42	0,04	9,83	0,43	0,04	9,30	0,33	0,04
LAG3	5,99	1,30	0,22	13,45	0,49	0,04	12,42	1,35	0,11
LAIR1	8,46	0,78	0,09	7,72	0,55	0,07	8,53	0,41	0,05
LAMP1	11,34	0,40	0,03	2,78	0,81	0,29	4,13	1,53	0,37
LAP3	10,46	0,79	0,08	8,91	0,32	0,04	8,65	0,40	0,05
LAYN	6,02	1,10	0,18	5,22	0,80	0,15	7,26	0,68	0,09
LCK	6,50	1,13	0,17	7,11	1,05	0,15	7,77	0,69	0,09
LCN2	7,53	1,50	0,20	8,03	0,22	0,03	8,64	0,67	0,08
LCP2	8,70	0,93	0,11	4,38	0,81	0,18	6,47	0,70	0,11
LDLR	7,31	0,75	0,10	7,85	0,59	0,07	8,65	0,50	0,06
LEF1	6,07	0,86	0,14	9,20	0,52	0,06	9,50	0,88	0,09
LGALS3	9,65	0,62	0,06	10,08	1,34	0,13	9,20	1,48	0,16
LHX6	5,42	1,34	0,25	5,72	0,66	0,12	6,91	0,85	0,12
LIF	7,14	1,00	0,14	3,02	1,50	0,50	3,56	1,32	0,37
LILRB1	7,51	1,01	0,13	3,98	1,40	0,35	6,61	1,72	0,26

LILRB2	8,38	1,34	0,16	2,93	1,29	0,44	5,22	1,93	0,37
LILRB4	7,84	1,05	0,13	8,72	0,60	0,07	8,98	0,56	0,06
LOX	7,20	1,23	0,17	8,82	0,26	0,03	7,95	1,15	0,15
LRP2	10,75	1,27	0,12	4,24	0,83	0,20	5,59	1,10	0,20
LRRC32	5,89	1,16	0,20	10,35	0,43	0,04	10,01	1,37	0,14
LST1	8,27	1,04	0,13	2,79	1,31	0,47	4,69	1,77	0,38
LTA	4,79	1,62	0,34	8,35	0,38	0,05	8,98	0,46	0,05
LTB	7,85	1,26	0,16	5,47	1,25	0,23	6,86	1,68	0,25
LTBR	8,68	0,31	0,04	5,18	1,02	0,20	7,20	1,16	0,16
LTF	8,91	1,66	0,19	6,82	0,77	0,11	6,77	0,99	0,15
LY96	7,44	0,98	0,13	4,43	0,53	0,12	5,40	0,68	0,13
LYVE1	8,68	1,07	0,12	13,62	0,34	0,03	13,76	1,78	0,13
MAF	8,30	0,55	0,07	4,35	0,44	0,10	5,04	0,67	0,13
MALL	4,06	1,75	0,43	6,88	0,35	0,05	7,82	0,71	0,09
MAP3K1	9,11	0,53	0,06	10,70	0,81	0,08	9,65	0,62	0,06
MAPK11	6,83	0,60	0,09	4,99	0,96	0,19	7,41	0,88	0,12
MAPK12	5,59	0,58	0,10	2,06	1,32	0,64	2,86	1,06	0,37
MAPK13	7,75	0,51	0,07	5,29	1,43	0,27	7,65	1,32	0,17
MAPK14	9,02	0,25	0,03	6,48	0,54	0,08	8,32	0,97	0,12
MAPK3	8,68	0,30	0,03	4,44	1,11	0,25	6,39	1,07	0,17
MAPK8	7,29	0,45	0,06	7,96	0,41	0,05	8,68	0,29	0,03
MARCH8,	6,53	0,33	0,05	4,08	1,18	0,29	5,89	2,19	0,37
MASP1	5,29	0,81	0,15	6,16	0,45	0,07	6,95	0,73	0,11
MASP2	3,34	2,06	0,62	7,62	0,38	0,05	7,28	0,90	0,12
MBP	8,13	0,34	0,04	5,70	0,91	0,16	7,78	0,94	0,12
MCAM	7,55	0,68	0,09	6,89	0,90	0,13	7,96	1,77	0,22
MCM6	7,33	0,52	0,07	4,72	1,26	0,27	5,06	1,23	0,24
MEF2C	8,24	0,61	0,07	3,03	1,00	0,33	4,45	1,33	0,30
MEGF11	6,01	1,22	0,20	6,54	1,07	0,16	8,03	0,99	0,12
MEOX1	4,57	1,97	0,43	7,13	0,38	0,05	8,30	0,62	0,07
MERTK	8,28	0,60	0,07	3,84	0,99	0,26	5,49	1,93	0,35
MET	8,84	0,60	0,07	6,87	0,41	0,06	7,82	0,55	0,07
MICA	8,06	0,51	0,06	3,95	1,04	0,26	6,28	1,01	0,16
MICB	8,20	0,68	0,08	6,87	1,12	0,16	8,32	1,05	0,13
MIF	11,51	0,63	0,05	3,00	1,23	0,41	3,79	1,12	0,30
MIR155HG	6,44	1,00	0,16	3,63	1,87	0,52	5,90	3,20	0,54
MME	10,18	1,09	0,11	7,33	0,60	0,08	8,74	0,67	0,08
MMP12	3,54	1,54	0,43	5,38	1,06	0,20	4,60	2,60	0,56
MMP14	8,57	0,74	0,09	4,74	1,00	0,21	6,69	1,59	0,24
MMP9	6,85	1,13	0,16	7,58	1,02	0,13	7,83	0,88	0,11
MMRN2	7,33	0,91	0,12	8,84	0,30	0,03	9,13	0,44	0,05
MPIG6B	3,40	1,72	0,50	13,03	0,69	0,05	11,80	1,19	0,10
MRC1	9,09	0,62	0,07	8,28	0,55	0,07	9,43	1,05	0,11
MS4A1	5,13	1,81	0,35	7,82	0,60	0,08	7,08	0,63	0,09
MS4A2	3,72	1,78	0,48	8,33	0,37	0,04	8,22	1,18	0,14
MS4A4A	9,00	1,05	0,12	9,43	0,36	0,04	9,19	0,47	0,05

MS4A6A	9,23	0,70	0,08	9,28	0,25	0,03	9,06	0,24	0,03
MS4A7	8,27	0,79	0,10	5,51	0,78	0,14	6,14	1,02	0,17
MT1A	6,61	1,22	0,18	7,42	0,43	0,06	8,13	0,76	0,09
MT2A	12,60	1,08	0,09	4,12	1,36	0,33	6,35	1,75	0,28
MTOR	8,21	0,39	0,05	2,05	1,24	0,60	3,27	1,45	0,44
MUC1	10,11	0,38	0,04	4,11	1,43	0,35	6,20	1,54	0,25
MX1	7,43	1,09	0,15	4,56	0,77	0,17	5,24	1,24	0,24
MX2	5,14	1,00	0,20	7,31	0,39	0,05	7,18	0,52	0,07
MYB	4,03	1,63	0,41	10,34	0,38	0,04	9,30	1,00	0,11
MYBL1	4,63	0,73	0,16	4,16	0,82	0,20	6,00	1,86	0,31
MYC	7,85	0,81	0,10	7,11	1,21	0,17	7,81	0,73	0,09
MYD88	9,14	0,60	0,07	7,94	1,30	0,16	8,89	1,05	0,12
MYL9	10,74	0,63	0,06	4,64	0,84	0,18	6,11	1,10	0,18
MYOM2	4,25	1,99	0,47	4,26	1,03	0,24	6,29	1,81	0,29
NCAM1	5,76	1,51	0,26	13,82	0,38	0,03	13,40	0,76	0,06
NCR1	4,47	0,91	0,20	8,34	0,82	0,10	8,19	0,44	0,05
NFAM1	5,56	1,27	0,23	6,36	0,48	0,08	6,63	1,09	0,16
NFATC1	6,47	0,72	0,11	4,59	1,18	0,26	6,33	1,92	0,30
NFATC2	7,70	0,73	0,10	8,31	0,37	0,04	8,70	0,67	0,08
NFIL3	7,77	0,70	0,09	2,74	1,04	0,38	4,04	1,37	0,34
NFKB1	7,28	0,34	0,05	8,40	0,49	0,06	8,49	0,40	0,05
NFKB2	8,90	0,57	0,06	2,91	1,62	0,56	4,91	2,50	0,51
NFKBIA	10,69	0,59	0,06	5,69	1,02	0,18	7,69	1,03	0,13
NFKBIZ	8,71	0,87	0,10	4,36	1,15	0,26	7,03	1,20	0,17
NKG7	8,91	1,09	0,12	4,58	0,97	0,21	6,94	0,83	0,12
NLRC5	8,34	0,82	0,10	4,69	2,01	0,43	4,15	1,89	0,46
NLRP3	5,98	0,84	0,14	6,94	0,52	0,07	6,63	0,80	0,12
NNMT	8,21	1,39	0,17	6,79	2,97	0,44	8,63	3,58	0,42
NOD1	6,73	0,53	0,08	2,93	1,37	0,47	5,72	1,55	0,27
NOD2	5,63	0,97	0,17	4,38	1,09	0,25	6,50	0,68	0,11
NOS2	5,10	1,35	0,27	8,49	0,51	0,06	9,42	0,44	0,05
NOS3	7,73	0,84	0,11	10,65	0,61	0,06	10,64	1,01	0,10
NOTCH1	7,95	0,56	0,07	7,11	0,56	0,08	7,61	0,89	0,12
NOTCH2	9,67	0,50	0,05	7,32	0,52	0,07	8,48	0,51	0,06
NOX4	9,37	0,83	0,09	5,01	1,08	0,22	6,06	0,56	0,09
NPDC1	8,79	0,59	0,07	12,93	0,25	0,02	12,05	1,20	0,10
NPHS1	7,17	0,91	0,13	6,52	0,67	0,10	8,08	0,63	0,08
NPHS2	7,62	1,12	0,15	7,66	0,25	0,03	7,63	0,47	0,06
NPPA	2,22	2,44	1,10	10,30	0,21	0,02	11,04	0,70	0,06
NPPB	3,32	2,12	0,64	2,92	1,26	0,43	4,67	2,43	0,52
NR4A1	6,59	1,51	0,23	5,92	0,56	0,10	6,59	1,09	0,17
OASL	4,45	2,55	0,57	8,01	0,25	0,03	7,94	0,37	0,05
OR2I1P	4,42	1,56	0,35	10,87	0,80	0,07	9,66	1,67	0,17
OSMR	8,10	0,73	0,09	8,08	0,45	0,06	8,67	0,38	0,04
P2RX4	7,59	0,65	0,09	3,75	1,70	0,45	6,08	1,98	0,33
PADI4	3,50	1,36	0,39	7,16	0,31	0,04	6,86	0,82	0,12

PALMD	5,51	1,21	0,22	3,77	0,67	0,18	4,31	0,98	0,23
PAX5	4,22	1,99	0,47	2,84	1,34	0,47	4,29	1,26	0,29
PDCD1	3,46	1,82	0,53	3,40	1,65	0,48	6,55	1,44	0,22
PDCD1LG2	6,31	0,72	0,11	5,91	0,66	0,11	7,43	0,87	0,12
PDGFA	7,32	0,52	0,07	4,92	0,94	0,19	7,33	0,66	0,09
PDGFRB	9,36	0,55	0,06	3,82	1,60	0,42	6,20	1,02	0,16
PDPN	5,08	1,25	0,25	6,39	0,85	0,13	7,62	0,73	0,10
PECAM1	9,96	0,88	0,09	8,29	2,06	0,25	9,34	3,13	0,34
PF4	4,56	1,81	0,40	4,17	1,05	0,25	6,85	1,24	0,18
PHEX	3,85	1,17	0,30	8,19	1,41	0,17	7,91	1,51	0,19
PIK3CD	7,88	0,50	0,06	9,93	0,35	0,04	9,04	0,99	0,11
PIK3CG	7,11	0,78	0,11	3,52	1,55	0,44	6,20	2,22	0,36
PIN1	7,79	0,20	0,03	5,46	0,85	0,16	6,63	1,35	0,20
PLA1A	7,61	1,15	0,15	3,55	1,14	0,32	5,99	1,18	0,20
PLAAT4	10,71	0,98	0,09	6,17	0,76	0,12	8,21	0,49	0,06
PLAT	8,92	0,68	0,08	6,92	0,65	0,09	8,21	1,33	0,16
PLAU	8,57	0,80	0,09	10,43	0,47	0,05	11,23	0,53	0,05
PLAUR	6,09	1,34	0,22	6,21	0,43	0,07	7,24	0,97	0,13
PLK2	7,75	0,64	0,08	4,38	1,15	0,26	7,12	1,04	0,15
PNOC	4,07	2,10	0,52	6,63	0,74	0,11	8,25	0,52	0,06
POU2AF1	4,81	2,17	0,45	6,18	0,87	0,14	6,32	0,93	0,15
PPBP	3,70	1,48	0,40	8,76	0,37	0,04	8,77	0,37	0,04
PPM1F	7,55	0,40	0,05	3,77	1,08	0,29	5,91	1,52	0,26
PPP3CA	8,61	0,33	0,04	9,06	0,15	0,02	8,95	0,24	0,03
PRDM1	6,73	1,03	0,15	9,27	0,84	0,09	9,20	0,81	0,09
PRF1	6,50	1,05	0,16	6,90	0,66	0,10	8,53	0,45	0,05
PROX1	4,97	2,05	0,41	3,45	1,52	0,44	5,69	2,30	0,40
PSEN1	8,88	0,26	0,03	8,80	0,37	0,04	8,92	0,24	0,03
PSMB10	8,75	0,83	0,09	5,30	0,64	0,12	6,09	1,12	0,18
PSMB8	9,95	0,76	0,08	7,51	0,43	0,06	7,41	0,56	0,08
PSMB9	9,56	0,94	0,10	9,91	0,30	0,03	10,34	0,86	0,08
PSME1	10,58	0,53	0,05	5,43	1,14	0,21	7,62	0,87	0,11
PSME2	10,97	0,69	0,06	5,15	1,08	0,21	7,82	0,63	0,08
PSTPIP1	6,20	0,93	0,15	8,69	1,87	0,22	10,46	2,21	0,21
PTGER4	8,15	0,60	0,07	2,16	1,21	0,56	2,85	1,25	0,44
PTGS2	5,64	1,49	0,26	5,52	1,42	0,26	7,10	1,47	0,21
PTPN2	7,90	0,36	0,05	3,93	1,25	0,32	7,03	1,11	0,16
PTPN22	6,15	0,94	0,15	7,27	0,25	0,03	7,61	0,90	0,12
PTPN6	8,60	0,56	0,06	7,13	0,95	0,13	8,65	0,89	0,10
PTPN7	6,03	0,96	0,16	5,14	0,68	0,13	5,49	1,33	0,24
PTPRC	10,06	0,90	0,09	2,65	1,17	0,44	3,77	1,19	0,32
PTPRO	7,33	0,88	0,12	8,06	0,23	0,03	8,15	0,18	0,02
PTX3	6,33	1,90	0,30	8,31	0,53	0,06	8,39	0,63	0,08
PVR	7,81	0,51	0,07	5,96	0,79	0,13	7,38	1,48	0,20
RAB40C	8,11	0,27	0,03	6,55	0,70	0,11	7,55	1,07	0,14
RAF1	9,69	0,21	0,02	10,22	0,49	0,05	10,14	0,71	0,07

RAG2	2,76	2,17	0,79	4,04	0,91	0,22	5,78	1,12	0,19
RAMP3	6,12	0,88	0,14	6,95	0,58	0,08	7,60	0,54	0,07
RAPGEF5	8,76	0,51	0,06	5,26	0,84	0,16	7,12	0,54	0,08
RARRES1	8,06	1,15	0,14	6,53	0,81	0,12	8,33	0,61	0,07
RASIP1	7,20	0,65	0,09	4,47	0,96	0,21	6,76	0,90	0,13
RASSF9	6,76	0,88	0,13	8,29	0,23	0,03	7,69	0,83	0,11
REL	7,29	0,44	0,06	6,66	0,54	0,08	7,43	0,66	0,09
RELA	7,45	0,50	0,07	7,24	0,82	0,11	8,86	0,63	0,07
RELB	7,49	0,82	0,11	7,07	0,75	0,11	8,80	0,60	0,07
RGN	8,62	1,01	0,12	11,35	0,22	0,02	11,02	0,70	0,06
RGS5	10,07	0,83	0,08	8,52	1,01	0,12	10,29	1,41	0,14
RHOJ	6,66	0,84	0,13	11,62	0,57	0,05	12,32	1,20	0,10
RHOU	7,76	0,51	0,07	10,80	0,45	0,04	9,63	1,40	0,15
RNF149	9,20	0,35	0,04	7,51	0,39	0,05	7,44	0,47	0,06
ROBO4	8,23	0,58	0,07	8,44	0,72	0,08	9,94	1,18	0,12
RORA	8,49	0,37	0,04	9,72	0,62	0,06	8,63	0,99	0,12
RORC	7,05	0,74	0,11	6,20	0,56	0,09	7,22	1,25	0,17
RPL19	13,55	0,36	0,03	5,27	0,92	0,18	6,52	1,37	0,21
RPS6	13,43	0,49	0,04	8,15	0,51	0,06	9,37	0,73	0,08
RPS6KB1	8,29	0,30	0,04	8,68	0,55	0,06	8,07	0,39	0,05
RTN4	11,07	0,41	0,04	7,39	0,37	0,05	8,20	0,51	0,06
RUNX1	7,34	0,93	0,13	7,84	1,03	0,13	8,91	0,97	0,11
RXRA	9,44	0,36	0,04	5,02	1,50	0,30	6,77	1,12	0,17
S100A12	5,19	1,58	0,30	11,60	0,68	0,06	10,11	0,87	0,09
S100A8	8,43	1,84	0,22	7,18	0,47	0,07	7,66	0,56	0,07
S100A9	9,78	1,66	0,17	7,33	0,37	0,05	7,73	0,37	0,05
S100B	5,32	1,36	0,26	4,43	1,14	0,26	7,11	1,33	0,19
S1PR1	6,47	0,89	0,14	6,48	0,57	0,09	7,29	0,96	0,13
SAMHD1	10,14	0,63	0,06	11,94	0,52	0,04	10,57	1,29	0,12
SCGB1A1	3,50	2,02	0,58	4,84	1,06	0,22	7,39	0,69	0,09
SDC1	9,41	0,72	0,08	5,31	1,71	0,32	7,79	1,14	0,15
SELE	5,20	1,45	0,28	7,86	0,51	0,07	8,32	0,51	0,06
SELL	5,78	1,00	0,17	2,78	1,03	0,37	5,05	1,29	0,26
SELP	5,78	1,12	0,19	8,39	0,24	0,03	8,45	0,32	0,04
SELPLG	7,13	0,78	0,11	2,36	1,05	0,44	3,31	0,95	0,29
SEMA7A	5,18	1,11	0,21	8,31	0,84	0,10	9,30	0,97	0,10
SERINC5	8,18	0,43	0,05	4,79	1,12	0,23	6,67	1,03	0,16
SERPINA3	9,57	1,65	0,17	6,70	0,38	0,06	6,94	0,76	0,11
SERPINE1	9,39	1,98	0,21	7,33	0,41	0,06	7,95	0,58	0,07
SERPING1	10,99	0,82	0,07	5,85	0,79	0,14	7,72	0,60	0,08
SERTAD1	6,99	0,57	0,08	2,17	2,26	1,04	5,21	2,55	0,49
SFTPA2	3,84	2,17	0,56	3,19	1,54	0,48	5,20	2,52	0,49
SFTPB	3,18	1,44	0,45	8,57	0,24	0,03	8,76	0,26	0,03
SFTPC	3,45	2,01	0,58	6,98	0,70	0,10	8,59	0,85	0,10
SFTPD	4,31	1,56	0,36	6,05	0,73	0,12	6,87	1,20	0,17
SH2D1A	5,15	0,98	0,19	7,53	1,21	0,16	8,23	0,76	0,09

SH2D1B	5,38	1,54	0,29	4,25	1,52	0,36	6,64	1,54	0,23
SHROOM3	7,67	0,31	0,04	4,46	0,78	0,17	6,41	0,84	0,13
SIGIRR	8,42	0,57	0,07	2,12	1,39	0,66	4,56	2,00	0,44
SIGLEC5	4,93	1,46	0,30	6,19	0,98	0,16	8,64	0,73	0,08
SIRPG	4,77	1,21	0,25	10,41	0,61	0,06	10,38	1,36	0,13
SKI	8,04	0,45	0,06	7,38	0,82	0,11	8,07	0,57	0,07
SLA	7,97	0,67	0,08	8,97	0,38	0,04	8,87	0,37	0,04
SLAMF6	6,68	1,17	0,18	3,69	1,01	0,27	5,71	1,65	0,29
SLAMF7	7,89	1,74	0,22	6,31	0,88	0,14	7,38	0,55	0,08
SLAMF8	7,62	1,12	0,15	7,88	0,41	0,05	8,07	0,22	0,03
SLC11A1	5,64	1,36	0,24	6,36	0,80	0,13	5,52	0,70	0,13
SLC12A3	9,45	1,38	0,15	7,15	1,08	0,15	8,18	0,83	0,10
SLC19A3	5,70	0,90	0,16	11,16	0,46	0,04	12,08	1,51	0,13
SLC22A2	10,51	1,28	0,12	4,49	1,10	0,25	6,46	1,72	0,27
SLC25A15	5,70	0,64	0,11	7,61	0,20	0,03	7,91	0,32	0,04
SLC4A1	5,91	1,33	0,23	7,90	0,71	0,09	9,96	1,35	0,14
SLPI	8,01	1,73	0,22	5,66	1,12	0,20	8,04	0,99	0,12
SMAD2	10,03	0,36	0,04	6,19	0,71	0,11	7,65	0,71	0,09
SMAD3	9,17	0,33	0,04	11,20	0,32	0,03	10,37	0,80	0,08
SMAD4	8,60	0,27	0,03	5,24	1,07	0,20	7,89	0,93	0,12
SMAD5	7,94	0,29	0,04	3,50	1,25	0,36	5,67	1,64	0,29
SMARCA4	9,08	0,29	0,03	8,77	0,96	0,11	10,29	1,42	0,14
SOCS1	7,35	0,82	0,11	5,92	0,69	0,12	7,45	0,97	0,13
SOCS3	7,78	1,17	0,15	3,95	1,22	0,31	6,51	2,13	0,33
SOD2	12,27	1,11	0,09	4,08	0,85	0,21	5,21	1,02	0,20
SOST	5,25	1,24	0,24	7,62	0,38	0,05	9,03	0,70	0,08
SOX7	5,31	1,00	0,19	7,85	0,43	0,05	8,75	0,52	0,06
SP100	8,77	0,44	0,05	15,65	0,39	0,03	14,89	1,08	0,07
SP140	6,98	1,17	0,17	10,72	0,63	0,06	12,15	1,00	0,08
SPIB	4,76	1,73	0,36	10,20	0,39	0,04	8,89	1,61	0,18
SPRY4	8,38	0,55	0,07	6,89	0,56	0,08	8,75	0,79	0,09
SRC	8,25	0,23	0,03	9,02	0,65	0,07	9,17	1,52	0,17
ST5	7,14	0,46	0,06	6,93	0,73	0,11	8,04	0,85	0,11
ST8SIA4	7,95	0,69	0,09	10,44	0,60	0,06	10,50	0,70	0,07
STAT1	10,69	0,92	0,09	6,77	1,84	0,27	8,99	1,24	0,14
STAT3	10,77	0,38	0,04	9,19	1,90	0,21	10,55	2,33	0,22
STAT4	6,69	0,84	0,13	4,73	0,72	0,15	6,29	0,94	0,15
STAT5A	8,36	0,41	0,05	7,10	0,39	0,06	8,18	1,28	0,16
STAT5B	8,54	0,23	0,03	8,79	0,22	0,03	7,86	1,37	0,17
STAT6	10,30	0,27	0,03	9,13	0,30	0,03	9,07	1,17	0,13
SYK	8,32	0,49	0,06	7,24	0,45	0,06	6,22	1,10	0,18
TANK	8,91	0,31	0,04	6,75	0,28	0,04	6,14	0,79	0,13
TAP1	8,64	1,06	0,12	8,79	1,18	0,13	8,61	1,53	0,18
TAP2	9,04	0,94	0,10	2,73	1,64	0,60	5,11	2,81	0,55
TAPBP	9,94	0,79	0,08	6,49	1,51	0,23	8,26	1,47	0,18
TBK1	7,90	0,25	0,03	7,78	0,75	0,10	8,33	0,47	0,06

TBX21	5,77	1,35	0,23	3,00	1,26	0,42	5,71	1,88	0,33
TCF7	6,39	1,05	0,16	2,82	1,00	0,35	4,57	0,95	0,21
TCL1A	3,93	1,91	0,49	6,48	0,44	0,07	7,36	0,49	0,07
TEK	7,62	0,80	0,10	11,57	0,23	0,02	10,62	0,91	0,09
TFF3	5,00	0,98	0,20	8,22	0,68	0,08	8,10	0,69	0,09
TFRC	8,68	0,55	0,06	8,27	0,76	0,09	9,07	0,92	0,10
TGFB1	9,64	0,60	0,06	8,08	0,23	0,03	8,44	0,50	0,06
TGFB2	5,80	1,02	0,18	7,90	0,72	0,09	9,29	0,77	0,08
TGFBI	9,62	0,83	0,09	7,49	0,66	0,09	8,26	0,63	0,08
TGFBR1	9,12	0,46	0,05	3,54	1,21	0,34	5,31	1,27	0,24
TGFBR2	10,20	0,45	0,04	4,16	1,12	0,27	6,74	0,87	0,13
TGIF1	8,06	0,51	0,06	3,64	1,26	0,35	5,72	1,17	0,21
THBD	7,55	0,73	0,10	8,23	0,82	0,10	8,63	0,61	0,07
THBS1	10,75	0,93	0,09	3,69	0,77	0,21	3,63	0,99	0,27
THEMIS	5,93	1,21	0,20	3,57	1,30	0,36	5,40	2,14	0,40
TIGIT	5,36	1,55	0,29	3,38	1,09	0,32	5,20	1,00	0,19
TIMP1	11,19	1,38	0,12	7,22	0,54	0,07	7,64	0,69	0,09
TIPARP	7,98	0,65	0,08	9,02	0,76	0,08	9,54	0,75	0,08
TLR2	8,16	0,88	0,11	6,09	0,58	0,09	6,75	0,62	0,09
TLR3	7,12	0,44	0,06	5,27	1,36	0,26	7,04	1,17	0,17
TLR4	8,96	0,50	0,06	7,72	0,23	0,03	8,18	0,40	0,05
TLR5	6,43	0,75	0,12	4,09	1,80	0,44	6,29	2,14	0,34
TLR7	5,32	1,00	0,19	7,94	0,67	0,08	8,01	0,68	0,08
TLR8	6,61	1,06	0,16	6,08	0,52	0,09	7,05	0,62	0,09
TLR9	5,14	1,61	0,31	4,23	1,06	0,25	5,95	2,04	0,34
TM4SF1	9,90	0,75	0,08	3,37	1,72	0,51	5,37	1,91	0,36
TM4SF18	8,30	0,84	0,10	1,94	1,43	0,74	4,37	1,89	0,43
TMEM178A	7,75	0,57	0,07	8,62	0,33	0,04	7,65	0,96	0,13
TNC	8,90	0,96	0,11	7,50	0,47	0,06	8,54	0,52	0,06
TNF	6,25	1,00	0,16	7,34	1,84	0,25	9,15	2,15	0,24
TNFAIP3	8,68	0,81	0,09	7,09	1,98	0,28	8,64	2,25	0,26
TNFAIP6	4,02	1,60	0,40	3,18	1,42	0,45	5,54	2,37	0,43
TNFRSF14	6,67	0,87	0,13	9,43	0,53	0,06	10,42	1,65	0,16
TNFRSF17	5,00	2,47	0,49	7,92	0,45	0,06	8,34	0,44	0,05
TNFRSF18	4,30	1,44	0,33	10,76	0,51	0,05	10,24	0,86	0,08
TNFRSF1A	10,03	0,33	0,03	11,15	0,58	0,05	9,90	0,63	0,06
TNFRSF1B	8,08	0,85	0,11	3,28	1,50	0,46	5,92	1,88	0,32
TNFRSF4	5,80	1,09	0,19	6,93	0,72	0,10	8,53	0,87	0,10
TNFRSF9	4,65	1,24	0,27	10,06	0,66	0,07	10,82	1,29	0,12
TNFSF10	10,68	1,14	0,11	9,84	0,94	0,10	9,95	0,86	0,09
TNFSF14	5,16	1,22	0,24	7,46	0,28	0,04	7,08	0,77	0,11
TNFSF18	2,99	1,53	0,51	9,76	1,03	0,11	9,86	1,31	0,13
TNFSF4	5,40	1,19	0,22	6,37	0,85	0,13	6,61	1,15	0,17
TNFSF8	6,42	1,07	0,17	4,05	1,37	0,34	5,85	1,87	0,32
TNFSF9	2,74	1,54	0,56	6,66	0,44	0,07	7,50	0,82	0,11
TOX2	5,14	1,40	0,27	1,91	1,17	0,61	2,95	0,79	0,27

TP53	8,03	0,30	0,04	6,20	0,70	0,11	7,38	0,84	0,11
TPMT	9,37	0,66	0,07	13,04	0,52	0,04	13,81	1,75	0,13
TPSAB1,B2	7,45	1,99	0,27	6,56	0,27	0,04	7,10	0,47	0,07
TRAF4	8,41	0,40	0,05	10,70	1,49	0,14	10,50	1,32	0,13
TRAF6	8,15	0,32	0,04	5,20	0,95	0,18	5,97	1,20	0,20
TRAT1	5,97	1,43	0,24	10,30	0,71	0,07	9,61	0,83	0,09
TRDC	6,17	1,30	0,21	2,60	1,13	0,43	4,48	1,54	0,34
TRDN	3,43	2,58	0,75	9,30	0,38	0,04	8,99	1,48	0,16
TRDV3	3,89	2,10	0,54	9,34	0,31	0,03	8,50	1,33	0,16
TREM1	5,00	1,71	0,34	8,24	0,31	0,04	8,17	0,91	0,11
TRIB1	9,25	0,59	0,06	5,12	1,09	0,21	7,14	1,09	0,15
TRIM22	7,74	0,72	0,09	4,33	1,05	0,24	5,34	1,35	0,25
TYK2	8,28	0,43	0,05	13,79	0,47	0,03	13,20	0,93	0,07
UMOD	11,70	2,31	0,20	3,95	1,07	0,27	6,05	1,77	0,29
VCAM1	10,39	1,00	0,10	3,91	1,37	0,35	6,08	1,35	0,22
VCAN	7,41	1,17	0,16	9,14	0,69	0,08	9,99	0,85	0,09
VEGFA	10,17	0,73	0,07	9,96	2,41	0,24	11,47	2,20	0,19
VEGFC	6,84	0,74	0,11	8,64	0,80	0,09	10,07	0,88	0,09
VMP1	9,28	0,42	0,04	8,47	0,22	0,03	8,66	0,26	0,03
VSIR	7,80	0,90	0,12	4,34	1,00	0,23	6,32	1,19	0,19
VWF	8,13	1,16	0,14	11,94	0,49	0,04	11,96	1,48	0,12
WARS	11,02	1,11	0,10	5,54	0,73	0,13	7,45	0,68	0,09
WNT9A	3,90	1,16	0,30	7,29	1,27	0,17	8,74	1,02	0,12
XAF1	8,69	1,03	0,12	8,46	0,67	0,08	8,24	0,49	0,06
XBP1	10,35	0,90	0,09	7,25	0,59	0,08	7,05	0,82	0,12
XCL1,2	5,76	1,10	0,19	3,24	0,97	0,30	3,28	0,85	0,26
ZAP70	7,61	1,17	0,15	6,83	0,81	0,12	7,35	1,28	0,17
ZEB1	7,66	0,57	0,07	2,69	1,05	0,39	3,34	0,74	0,22

Table 4S. Overview of genes in the NanoString nCounter® Elements™ panel with their corresponding mean, SD and CV in samples with AMR, NR or TCMR.

Gene	AMR			NR			TCMR		
	Mean	SD	CV	Mean	SD	CV	Mean	SD	CV
ACVRL1	7,14	0,54	0,08	6,27	0,41	0,07	7,09	0,73	0,10
AGR2	5,06	0,93	0,18	4,73	0,71	0,15	4,85	0,90	0,19
AGR3	3,39	1,05	0,31	2,62	0,62	0,24	2,98	1,42	0,48
ANXA1	8,66	0,63	0,07	7,53	0,75	0,10	8,49	0,85	0,10
APOBEC3A	2,22	1,79	0,81	0,96	1,29	1,34	3,05	2,71	0,89
CAV1	6,22	0,72	0,12	5,73	0,76	0,13	6,08	0,99	0,16
CCL3	5,65	1,30	0,23	3,69	1,05	0,28	6,21	1,68	0,27
CCL4	6,76	1,37	0,20	4,13	1,14	0,27	6,59	1,28	0,19
CD160	3,35	1,57	0,47	1,62	1,53	0,95	3,97	2,10	0,53
CD34	5,38	0,94	0,18	4,89	0,51	0,10	5,59	1,28	0,23
CD55	6,60	0,43	0,07	6,09	0,51	0,08	6,64	0,86	0,13
CD59	10,89	0,38	0,03	10,87	0,26	0,02	10,40	0,79	0,08
CD74	12,92	0,85	0,07	11,33	0,52	0,05	12,87	0,90	0,07
CDH13	5,18	1,16	0,22	4,64	0,79	0,17	5,61	1,48	0,26
CDH5	6,66	0,47	0,07	6,05	0,50	0,08	6,53	0,62	0,10
CETP	3,22	0,98	0,30	2,91	0,63	0,22	3,52	0,95	0,27
CFLAR	8,92	0,32	0,04	8,55	0,23	0,03	8,77	0,25	0,03
COL13A1	3,00	1,12	0,37	1,86	0,98	0,53	2,49	1,37	0,55
CRHBP	5,56	0,91	0,16	5,76	0,70	0,12	5,43	0,99	0,18
CRIP2	8,53	0,56	0,07	7,95	0,44	0,05	7,94	0,58	0,07
CX3CR1	5,67	1,18	0,21	4,21	0,82	0,19	5,76	1,17	0,20
CXCL10	6,72	2,22	0,33	3,89	1,51	0,39	6,72	2,28	0,34
CXCL11	6,89	2,41	0,35	3,71	1,34	0,36	7,31	2,19	0,30
DARC	3,97	1,54	0,39	2,50	1,25	0,50	4,43	1,30	0,29
ECSCR	4,77	0,67	0,14	3,92	0,59	0,15	4,53	0,42	0,09
ELTD1	6,31	0,59	0,09	5,70	0,55	0,10	6,39	0,79	0,12
EMP3	7,82	0,78	0,10	6,36	0,56	0,09	7,73	0,46	0,06
ENG	9,23	0,54	0,06	8,78	0,36	0,04	8,62	0,91	0,11
ERG	4,60	0,99	0,22	3,72	0,53	0,14	4,48	1,03	0,23
EVA1C	5,25	0,45	0,09	4,74	0,54	0,12	5,01	0,61	0,12
FCGR3A	7,92	1,15	0,15	5,73	1,00	0,18	7,88	1,24	0,16
FGFBP2	4,21	1,30	0,31	2,57	1,08	0,42	4,36	1,91	0,44
GATA3	7,36	0,74	0,10	7,03	0,35	0,05	7,37	0,88	0,12
GNG11	8,06	0,49	0,06	7,75	0,35	0,04	7,94	0,49	0,06
GNLY	6,34	0,98	0,16	4,42	0,99	0,22	6,98	1,12	0,16
HEG1	7,31	0,49	0,07	6,80	0,50	0,07	7,56	0,58	0,08
HSPA12B	5,18	1,16	0,22	4,11	0,50	0,12	5,14	1,57	0,31
ICAM2	6,66	0,67	0,10	5,62	0,55	0,10	6,61	0,83	0,13
IER5	6,79	0,63	0,09	5,86	0,50	0,09	7,25	0,61	0,08
IFI27	8,56	0,87	0,10	7,34	0,58	0,08	8,18	0,76	0,09

IFNG	3,07	1,89	0,62	1,23	1,22	0,99	4,48	1,84	0,41
IL18RAP	3,39	1,12	0,33	1,89	1,02	0,54	3,59	1,34	0,37
KLF4	6,01	0,90	0,15	5,45	0,80	0,15	6,46	0,89	0,14
KLRF1	2,60	1,24	0,47	1,52	1,26	0,83	2,40	1,29	0,54
LAYN	4,20	1,19	0,28	3,33	0,71	0,21	4,70	1,77	0,38
LDLR	5,98	0,78	0,13	5,74	0,87	0,15	6,25	0,88	0,14
LHX6	4,49	1,33	0,30	2,87	1,07	0,37	4,59	1,99	0,43
LST1	7,40	1,00	0,14	5,49	0,86	0,16	7,77	0,97	0,13
MALL	6,40	0,60	0,09	6,06	0,49	0,08	6,42	0,47	0,07
MCAM	7,05	0,66	0,09	6,30	0,64	0,10	6,95	0,52	0,08
MEOX1	2,70	1,77	0,66	1,97	1,50	0,76	4,01	2,26	0,56
MMRN2	6,90	0,84	0,12	6,20	0,41	0,07	6,54	0,79	0,12
MYBL1	3,17	0,66	0,21	2,05	0,74	0,36	3,37	0,59	0,18
NOS3	6,49	0,99	0,15	5,37	0,45	0,08	6,33	0,89	0,14
NPDC1	6,26	0,55	0,09	5,45	0,52	0,09	6,21	0,92	0,15
PALMD	5,00	1,00	0,20	4,60	0,92	0,20	5,40	1,64	0,30
PECAM1	8,87	0,64	0,07	8,10	0,48	0,06	8,21	0,86	0,10
PGM5	6,17	0,70	0,11	5,76	0,48	0,08	6,12	1,09	0,18
PLA1A	6,63	1,19	0,18	5,23	0,60	0,12	6,54	0,90	0,14
PLAT	9,28	0,69	0,07	8,49	0,56	0,07	8,54	0,41	0,05
PLK2	1,60	1,30	0,81	0,54	0,77	1,41	1,63	1,23	0,75
PPM1F	7,01	0,36	0,05	6,43	0,35	0,05	7,04	0,62	0,09
PSMB10	6,45	0,89	0,14	5,00	0,46	0,09	6,75	0,93	0,14
RAMP3	7,60	0,70	0,09	7,12	0,29	0,04	7,33	0,47	0,06
RAPGEF5	7,38	0,62	0,08	6,50	0,41	0,06	7,15	0,58	0,08
RASIP1	5,55	0,83	0,15	4,92	0,52	0,11	5,84	1,12	0,19
RASSF9	5,60	0,81	0,14	4,98	0,44	0,09	5,69	1,43	0,25
RHOJ	4,85	1,02	0,21	3,95	0,87	0,22	4,87	1,25	0,26
ROBO4	7,20	0,58	0,08	6,31	0,41	0,06	6,68	0,59	0,09
RPS6	12,36	0,37	0,03	12,50	0,26	0,02	12,25	0,62	0,05
RPS6KB1	7,06	0,42	0,06	6,70	0,28	0,04	7,24	0,69	0,10
S1PR1	6,08	0,65	0,11	5,31	0,53	0,10	6,30	0,94	0,15
S1PR5	3,88	1,31	0,34	2,02	1,11	0,55	4,26	1,76	0,41
SDR16C5	1,56	2,25	1,44	0,90	1,50	1,67	2,83	2,95	1,04
SELE	3,51	1,92	0,55	2,18	1,34	0,61	4,38	1,82	0,42
SELP	4,24	1,57	0,37	3,57	1,04	0,29	4,45	1,80	0,40
SH2D1B	3,75	1,48	0,39	2,11	1,22	0,58	4,27	2,12	0,50
SOST	2,78	1,50	0,54	2,08	1,19	0,57	3,05	2,13	0,70
SOX7	5,23	0,93	0,18	4,30	0,68	0,16	5,59	1,72	0,31
TBX21	4,06	1,21	0,30	2,32	1,12	0,48	4,62	1,80	0,39
TEK	6,00	0,65	0,11	5,74	0,40	0,07	5,13	1,07	0,21
TFF3	3,50	1,51	0,43	3,32	1,03	0,31	4,37	2,05	0,47
THBD	6,24	0,80	0,13	5,28	0,53	0,10	6,46	1,04	0,16
TM4SF1	6,67	0,62	0,09	6,42	0,38	0,06	6,54	0,81	0,12
TM4SF18	5,93	0,87	0,15	5,38	0,50	0,09	5,97	0,86	0,14
TNF	5,60	1,26	0,23	3,58	0,92	0,26	6,06	1,32	0,22

TRDC	4,16	1,34	0,32	2,19	0,95	0,43	4,05	0,94	0,23
TRIB1	8,30	0,54	0,07	8,05	0,53	0,07	7,99	0,38	0,05
VEGFC	4,30	0,95	0,22	3,24	0,85	0,26	4,32	0,85	0,20
VWF	6,60	1,12	0,17	5,80	1,03	0,18	6,34	1,06	0,17

Table 5S. Top 20 differentially expressed genes in samples with BLorTCMR compared to samples with NoRejection using the B-HOT panel.

Gene	FC (log2)	SE (log2)	Lower confidence limit (log2)	Upper confidence limit (log2)	FDRPV[a]	Annotation of transcripts
**CD70**	4.93	0.331	4.28	5.58	2.96e-21	T cell checkpoint signaling
**TLR9**	4.3	0.335	3.64	4.95	7.82e-18	Innate immune system, Toll-like receptor signaling
**PF4**	3.96	0.326	3.32	4.59	1.02e-16	Chemokine signaling, hematopoiesis,
**TOX2**	4.63	0.387	3.87	5.39	1.62e-16	Epigenetics & Transcription
**SH2D1B**	3.76	0.337	3.1	4.42	4.59e-15	Adaptive immune system, cytotoxicity
**GZMB**	4.15	0.389	3.39	4.91	2.91e-14	Apoptosis & cell cycle regulation, cytotoxicity
**CD8B**	4.42	0.416	3.6	5.23	3.54e-14	Adaptive immune system, cell-ECM interaction, hematopoiesis, T cell receptor signaling,
**NKG7**	4.46	0.434	3.6	5.31	1.28e-13	Cytotoxic cells
**IDO1**	5.04	0.497	4.07	6.02	1.92e-13	Metabolism
**CCL5**	4.23	0.422	3.41	5.06	2.88e-13	Chemokine signaling, cytosolic DNA sensing, hematopoiesis, NLR signaling, oxidative stress, TNF family signaling, Toll-like receptor signaling,
**CALHM6**	4.31	0.439	3.45	5.18	7.28e-13	Tissue homeostasis

**GZMA**	4.01	0.412	3.2	4.81	1.04e-12	Cytotoxic cells
**CXCL11**	4.84	0.505	3.85	5.83	1.68e-12	Chemokine signaling, Toll-like receptor signaling
**IKZF1**	4.23	0.45	3.35	5.11	3.8e-12	Hematopoiesis
**IL18BP**	4.2	0.449	3.32	5.08	4.14e-12	Cytokine signaling, oxidative stress
**SLAMF7**	4.26	0.458	3.36	5.15	5.46e-12	Adaptive immune system
**LHX6**	3.3	0.36	2.59	4	1.03e-11	Epigenetics, transcription
**CD3D**	4.09	0.45	3.2	4.97	1.32e-11	Adaptive immune system, hematopoiesis, T-cell receptor signaling
**CD96**	2.9	0.32	2.27	3.52	1.32e-11	Adaptive immune system
**MICB**	3.85	0.429	3.01	4.69	1.89e-11	Adaptive immune system, cytotoxicity, oxidative stress

Positive ratio means higher expression in samples with BLorTCMR. Negative ratio means higher expression in samples with NoRejection.

[a]FDR *p*-value was obtained from the adjusted *p*-value of FDR correction by Benjamini-Yekutieli method.

Table 6S. Top 20 differentially expressed genes in samples with AMR compared to samples with NoRejection using the B-HOT panel.

Gene	FC (log2)	SE (log2)	Lower confidence limit (log2)	Upper confidence limit (log2)	FDRPV[a]	Annotation of transcripts
**MS4A1**	3.85	0.314	3.24	4.47	1.57e-16	B cells, hematopoiesis
**CXCL11**	5.37	0.495	4.4	6.34	3.79e-14	Chemokine signaling, Toll-like receptor signaling
**GBP5**	5.6	0.527	4.56	6.63	7.13e-14	NLR signaling, type II interferon signaling
**SLAMF7**	4.6	0.449	3.72	5.48	2.85e-13	Adaptive immune system
**HLA-F**	4.53	0.447	3.66	5.41	3.57e-13	Adaptive immune system, cell-ECM interaction, MHC Class I antigen presentation
**IDO1**	4.75	0.487	3.8	5.7	1.76e-12	Metabolism
**CCL5**	3.87	0.413	3.06	4.68	9.23e-12	Chemokine signaling, cytosolic DNA sensing, hematopoiesis, NLR signaling, oxidative stress, TNF family signaling, Toll-like receptor signaling, type I and type II interferon signaling
**CXCL10**	4.47	0.48	3.53	5.41	9.29e-12	Chemokine signaling, cytosolic DNA sensing, Th17 mediated biology, TNF family signaling, Toll-like receptor signaling
**ZAP70**	4.83	0.519	3.81	5.85	9.29e-12	Adaptive immune system, cytotoxicity, hematopoiesis, NF-kappa B signaling, T-cell receptor signaling

**CXCL9**	4.84	0.524	3.82	5.87	1.1e-11	Chemokine signaling, Toll-like receptor signaling
**ISG20**	3.74	0.428	2.9	4.58	9.72e-11	Type I interferon signaling
**XAF1**	3.41	0.392	2.65	4.18	9.83e-11	Cytokine signaling, Type I interferon signaling,
**IRF1**	3.41	0.391	2.64	4.18	9.83e-11	Hematopoiesis, innate immune system, Type I and type II interferon signaling
**CALHM6**	3.74	0.431	2.9	4.59	9.93e-11	Tissue homeostasis
**PSMB9**	3.43	0.396	2.65	4.2	1.1e-10	Adaptive immune system, apoptosis & cell cycle regulation, B cell receptor signaling, innate immune system, metabolism, MHC Class I antigen presentation, NF-kappa, B signaling, T cell receptor signaling
**TAP2**	3.5	0.414	2.69	4.31	2.57e-10	Adaptive immune system, MHC Class I antigen presentation
**CIITA**	3.18	0.377	2.44	3.92	2.62e-10	Type II interferon signaling
**APOL1**	3.22	0.384	2.47	3.97	3.2e-10	Metabolism
**TAP1**	3.3	0.395	2.53	4.07	3.26e-10	Adaptive immune system, MHC Class I antigen presentation
**FGD2**	3.86	0.463	2.95	4.77	3.28e-10	Apoptosis & cell cycle regulation

Positive ratio means higher expression in samples with AMR. Negative ratio means higher expression in samples with NoRejection.
[a]FDR *p*-value was obtained from the adjusted *p*-value of FDR correction by Benjamini-Yekutieli method.

Table 7S. Top 20 differentially expressed genes in samples with AMR compared to samples with BLorTCMR using the B-HOT panel.

Gene	FC (log2)	SE (log2)	Lower confidence limit (log2)	Upper confidence limit (log2)	FDRPV[a]	Annotation of transcripts
**CD70**	-4.17	0.353	-4.86	-3.48	1.15e-15	T cell checkpoint signaling
**TOX2**	-4.06	0.365	-4.78	-3.35	1.22e-14	Epigenetics & Transcription
**PF4**	-3.57	0.425	-4.4	-2.74	1.84e-09	Chemokine signaling, hematopoiesis,
**SH2D1B**	-2.78	0.34	-3.45	-2.12	3.68e-09	Adaptive immune system, cytotoxicity
**TLR9**	-3.15	0.409	-3.95	-2.34	2.91e-08	Innate immune system, Toll-like receptor signaling
**TNFSF8**	-3.33	0.448	-4.21	-2.45	7.03e-08	Hematopoiesis, TNF family signaling
**CD8B**	-2.15	0.294	-2.72	-1.57	1.13e-07	Adaptive immune system, cell-ECM interaction, hematopoiesis, T cell receptor signaling,
**C9**	-3.5	0.494	-4.47	-2.53	2.65e-07	Complement system, innate immune system,
**ADORA2A**	-3.34	0.511	-4.34	-2.34	2.54e-06	T cell checkpoint signaling
**BMP2**	-2.48	0.394	-3.26	-1.71	6.62e-06	Cell-ECM interaction, TGF-beta signaling
**BMP7**	-2.36	0.379	-3.11	-1.62	8.04e-06	Cell-ECM interaction, oxidative stress, TGF-beta signaling
**MS4A1**[b]	1.88	0.306	1.28	2.48	1.11e-05	B cells, hematopoiesis

**LTA**	-3.2	0.524	-4.22	-2.17	1.21e-05	Cytokine signaling, NF-kappaB signaling, TNF family signaling,
**HNF1A**	-2.53	0.416	-3.35	-1.71	1.23e-05	Oxidative stress
**LHX6**	-2.54	0.443	-3.41	-1.67	4.98e-05	Epigenetics, transcription
**NOS2**	-2.67	0.469	-3.59	-1.76	5.37e-05	Innate immune system
**MIR155HG**	-2.17	0.393	-2.94	-1.4	0.000104	Hematopoiesis
**GZMB**	-2.17	0.396	-2.95	-1.4	0.000112	Apoptosis & cell cycle regulation, cytotoxicity
**ASB15**	-2.36	0.435	-3.21	-1.5	0.000144	Adaptive immune system, MHC Class I antigen presentation
**CD79A**	-2.84	0.528	-3.88	-1.81	0.000161	Adaptive immune system, B cell receptor signaling, hematopoiesis

Positive ratio means higher expression in samples with AMR. Negative ratio means higher expression in samples with BLorTCMR.

[a] FDR *p*-value was obtained from the adjusted *p*-value of FDR correction by Benjamini-Yekutieli method.

[b] Only gene in the top 20 of differentially expressed genes with higher expression levels in AMR samples.

Table 8S. Top 20 differentially expressed genes in samples with BLorTCMR compared to samples with NoRejection using the NanoString nCounter® Elements™ panel.

Gene	FC (log2)	SE (log2)	Lower confidence limit (log2)	Upper confidence limit (log2)	FDRPV[a]	Annotation of transcripts
**SOX7**	4.92	0.546	3.85	5.99	8.41e-11	Epigenetics & transcription
**CCL3**	5.28	0.591	4.12	6.43	8.41e-11	Chemokine signaling
**CXCL11**	4.79	0.57	3.68	5.91	5.11e-10	Chemokine signaling, Toll-like receptor signaling
**TNF**	3.43	0.434	2.58	4.28	3.36e-09	Apoptosis & cell cycle regulation, cytotoxicity, hematopoiesis, MAPK, mTOR, NF-kappaB signaling, NLR signaling, oxidative stress, T cell receptor signaling, TGF-beta signaling, Th17 differentiation, Th17 mediated biology, TNF family signaling, Toll-like receptor signaling
**RASSF9**	3.73	0.481	2.79	4.68	3.91e-09	Tissue homeostasis
**CXCL10**	4.61	0.595	3.45	5.78	3.91e-09	Chemokine signaling, cytosolic DNA sensing, Th17 mediated biology, TNF family signaling, Toll-like receptor signaling
**LST1**	3.73	0.484	2.78	4.68	4.15e-09	Type II interferon signaling
**CD160**	3.79	0.509	2.79	4.79	1.14e-08	Adaptive immune system
**HSPA12B**	3.86	0.521	2.84	4.88	1.15e-08	Tissue homeostasis
**CDH13**	3.59	0.491	2.63	4.55	1.52e-08	Cell-ECM interaction

**PSMB10**	2.48	0.355	1.78	3.17	5.61e-08	Adaptive immune system, apoptosis & cell cycle regulation, B cell receptor signaling, innate immune system, metabolism, MHC Class I antigen presentation, NF-kappa B signaling, T cell receptor signaling
**THBD**	2.67	0.386	1.91	3.43	6.76e-08	Complement system
**GNLY**	2.63	0.397	1.85	3.41	2.23e-07	Cytotoxic cells, innate immune system
**CCL4**	2.78	0.46	1.88	3.68	2.05e-06	Chemokine signaling, cytosolic DNA sensing, NF-kappaB signaling, Toll-like receptor signaling
**IER5**	2.19	0.363	1.48	2.9	2.05e-06	Tissue homeostasis
**RASIP1**	2.29	0.381	1.55	3.04	2.05e-06	Angiogenesis
**S1PR1**	2.06	0.349	1.37	2.74	3.06e-06	Cytokine signaling
**CD74**	2.72	0.468	1.8	3.63	4.34e-06	Adaptive immune system, hematopoiesis, MHC Class II presentation
**FGFBP2**	3.04	0.527	2	4.07	5.01e-06	Cytotoxicity
**SH2D1B**	3.47	0.613	2.26	4.67	7.34e-06	Adaptive immune system, cytotoxicity

Positive ratio means higher expression in samples with BLorTCMR. Negative ratio means higher expression in samples without rejection.

[a]FDR *p*-value was obtained from the adjusted *p*-value of FDR correction by Benjamini-Yekutieli method.

Table 9S. Top 20 differentially expressed genes in samples with AMR compared to samples with NoRejection using the NanoString nCounter® Elements™ panel.

Gene	FC (log2)	SE (log2)	Lower confidence limit (log2)	Upper confidence limit (log2)	FDRPV[a]	Annotation of transcripts
**CXCL11**	5.75	0.528	4.72	6.79	5.23e-14	Chemokine signaling, Toll-like receptor signaling
**CXCL10**	5.26	0.551	4.18	6.34	6.39e-12	Chemokine signaling, cytosolic DNA sensing, Th17 mediated biology, TNF family signaling, Toll-like receptor signaling
**CCL4**	3.84	0.426	3	4.67	4.21e-11	Chemokine signaling, cytosolic DNA sensing, NF-kappaB signaling, Toll-like receptor signaling
**LST1**	3.49	0.448	2.62	4.37	4.97e-09	Type II interferon signaling
**EMP3**	2.92	0.41	2.12	3.72	6.8e-08	Cell-ECM interaction
**NOS3**	2.85	0.403	2.06	3.64	6.8e-08	Angiogenesis, innate immune system, metabolism, oxidative stress
**PSMB10**	2.26	0.329	1.62	2.91	1.13e-07	Adaptive immune system, apoptosis & cell cycle regulation, B cell receptor signaling, innate immune system, metabolism, MHC Class I antigen presentation, NF-kappaB signaling, T cell receptor signaling

**CD74**	2.98	0.433	2.13	3.83	1.13e-07	Adaptive immune system, hematopoiesis, MHC Class II presentation
**PLA1A**	2.65	0.388	1.89	3.41	1.13e-07	Metabolism
**IFI27**	2.76	0.404	1.97	3.55	1.13e-07	IFN gamma signaling, cytokine signaling
**FCGR3A**	3.42	0.509	2.42	4.41	1.71e-07	Neutrophils
**CCL3**	3.62	0.548	2.55	4.69	2.42e-07	Chemokine signaling
**CX3CR1**	2.19	0.357	1.49	2.89	1.53e-06	Chemokine signaling
**ICAM2**	2.08	0.351	1.39	2.77	3.29e-06	Adaptive immune system, cell-ECM interaction, cytotoxicity, innate immune system
**GNLY**	2.08	0.369	1.36	2.81	9.27e-06	Cytotoxic cells, innate immune system
**TNF**	2.26	0.404	1.47	3.05	1.13e-05	Apoptosis & cell cycle regulation, cytotoxicity, hematopoiesis, MAPK, mTOR, NF-kappaB signaling, NLR signaling, oxidative stress, T cell receptor signaling, TGF-beta signaling, Th17 differentiation, Th17 mediated biology, TNF family signaling, Toll-like receptor signaling
**RAPGEF5**	1.9	0.342	1.23	2.57	1.23e-05	MAPK
**ROBO4**	1.59	0.295	1.01	2.17	2.25e-05	Angiogenesis

**CD160**	2.56	0.493	1.59	3.52	4.58e-05	Adaptive immune system
**KLF4**	2.33	0.473	1.41	3.26	1.14e-04	Hematopoiesis, oxidative stress

Positive ratio means higher expression in samples with AMR. Negative ratio means higher expression in samples with NoRejection.

[a]FDR *p*-value was obtained from the adjusted *p*-value of FDR correction by Benjamini-Yekutieli method.

Table 10S. Top 20 differentially expressed genes in samples with AMR compared to samples with BLorTCMR using the NanoString nCounter® Elements™ panel.

Gene	FC (log2)	SE (log2)	Lower confidence limit (log2)	Upper confidence limit (log2)	FDRPV[a]	Annotation of transcripts
**SOX7**	-3.41	0.556	-4.50	-2.32	2.04e-05	Epigenetics & transcription
**RASSF9**	-2.48	0.491	-3.44	-1.52	7.18 e-04	Tissue homeostasis
**CDH13**	-2.32	0.501	-3.30	-1.34	2.35 e-03	Cell-ECM interaction
**HSPA12B**	-2.27	0.531	-3.31	-1.23	6.42 e-03	Tissue homeostasis
**VWF**	1.76	0.520	-0.30	2.78	0.101	Cell-ECM interaction, complement system
**CD160**	-1.23	0.408	-2.03	-0.43	0.250	Adaptive immune system
**ROBO4**	0.96	0.326	0.32	1.60	0.270	Angiogenesis
**THBD**	-1.14	0.394	-1.91	-0.37	0.270	Complement system
**CCL3**	-1.66	0.600	-2.83	-0.48	0.324	Chemokine signaling
**IER5**	-1.02	0.371	-1.75	-0.29	0.324	Tissue homeostasis
**IFI27**	1.21	0.446	0.34	2.08	0.326	IFN-gamma signaling, cytokine signaling
**TNF**	-1.17	0.439	-2.03	-0.31	0.343	Apoptosis & cell cycle regulation, cytotoxicity, hematopoiesis, MAPK, mTOR, NF-kappaB signaling, NLR signaling, oxidative stress, T cell receptor signaling, TGF-beta signaling, Th17 differentiation, Th17 mediated biology, TNF

						family signaling, Toll-like receptor signaling
**TEK**	0.93	0.358	0.22	1.63	0.385	Hematopoiesis, MAPK
**EMP3**	1.16	0.453	0.27	2.04	0.385	Cell-ECM interaction
**CRIP2**	1.12	0.442	0.25	1.99	0.385	Hematopoiesis
**TRIB1**	1.08	0.441	0.21	1.94	0.452	Hematopoiesis
**RASIP1**	-0.94	0.388	-1.70	-0.18	0.452	Angiogenesis
**MEOX1**	-1.50	0.648	-2.77	-0.24	0.525	Hematopoiesis
**NOS3**	1.03	0.444	0.16	1.90	0.525	Angiogenesis, innate immune system, metabolism, oxidative stress
**CCL4**	1.06	0.467	0.14	1.97	0.565	Chemokine signaling, cytosolic DNA sensing, NF-kappaB signaling, Toll-like receptor signaling

Positive ratio means higher expression in samples with AMR. Negative ratio means higher expression in samples with BLorTCMR.

Only the first four genes (SOX7, RASSF9, CDH13, HSPA12B) showed significantly higher expression levels in BLorTCMR samples. There were no genes with significantly higher expression levels in AMR samples.

[a]FDR *p*-value was obtained from the adjusted *p*-value of FDR correction by Benjamini-Yekutieli method.

Table 11S. Confusion matrix of the B-HOT model performances during cross-validation.

		True Class	
		AMR	no AMR
**Predicted Class**	AMR	23	0
	No AMR	5	57

AMR; antibody-mediated rejection

Table 12S. Confusion matrix of the Elements model performances during cross-validation.

		True Class	
		AMR	no AMR
**Predicted Class**	AMR	12	1
	No AMR	13	46

AMR; antibody-mediated rejection

Table 13S. Confusion matrix of the Elements model performances when tested on the B-HOT panel analysis.

		True Class	
		AMR	no AMR
**Predicted Class**	AMR	16	2
	No AMR	12	55

AMR; antibody-mediated rejection
